# Copper Depletion Nanoparticles Potentiate Cancer Immunotherapy by Avoiding Innate and Adaptive Immune Resistance

**DOI:** 10.1002/advs.202524150

**Published:** 2026-04-02

**Authors:** Zaigang Zhou, Ke Li, Xuelan Li, Lei Yi, Zhengxiang Wang, Huan Ding, Sheng Wu, Feiyu Liu, Yuan Li, Rui Cheng, Jianliang Shen

**Affiliations:** ^1^ Zhejiang Key Laboratory of Ophthalmic Drug Discovery and Medical Device Research Eye Hospital Wenzhou Medical University Wenzhou Zhejiang China; ^2^ Department of Urology The Second Xiangya Hospital Central South University Changsha Hunan China; ^3^ Department of Clinical Nursing Xiangya Hospital Central South University Changsha Hunan China; ^4^ Department of Hepatobiliary Surgery Fujian Medical University Union Hospital Fuzhou Fujian China; ^5^ Zhejiang Engineering Research Center for Tissue Repair Materials Wenzhou Institute University of Chinese Academy of Sciences Wenzhou Zhejiang China

**Keywords:** cluster of differentiation 47, copper depletion nanoparticles, immune resistance, mitochondria, programmed death ligand 1

## Abstract

Currently, cluster of differentiation 47 (CD47) and programmed death ligand 1 (PD‐L1) targeted bispecific antibodies have been widely studied in clinical trials to overcome innate and adaptive immune resistance simultaneously. However, the excessive immune‐related adverse events caused by the on‐target off‐tumor immune‐toxicity cast a shadow over their future clinical usage. Thus, how to safely, effectively, and selectively regulate CD47 and PD‐L1 in tumors at the same time is still a difficult issue to solve. Here, we developed a mitochondria‐targeted copper‐withdrawal nanoparticle CYN‐CDA@Alb to more efficaciously depress CD47 and PD‐L1 expression (only 1/50 dosage of common copper chelators), on account of the depression of mitochondria/Adenosine 5’‐monophosphate‐activated protein kinase (AMPK)/c‐MYC signal pathway. By doing this, CYN‐CDA@Alb reverses immune resistance by increasing T cell killing capacity and macrophage phagocytosis ability to tumor cells, leading to the following depressed tumor metastasis and slowed tumor growth. Moreover, CYN‐CDA@Alb also avoids the usually increased innate and adaptive immune resistance after radiotherapy by depressing CD47 and PD‐L1. Our findings altogether suggest the potential usage of copper ion‐depleting nanoparticles as substitutes for CD47/PD‐L1 bispecific antibodies to simultaneously overcome innate and adaptive immune‐resistance.

## Graphical abstract

A mitochondria‐targeted copper depletion nanoplatform (CYN‐CDA@Alb) was developed to selectively disrupt tumor mitochondria copper, which then reprogrammed the tumor immune microenvironment by depressing PD‐L1 and CD47 expression simultaneously. By doing this, CYN‐CDA@Alb reversed radiotherapy‐induced immune tolerance, showing the potential usage of copper ion‐depleting nanoparticles as substitutes for CD47/PD‐L1 bispecific antibodies to simultaneously overcome innate and adaptive immune‐resistance.

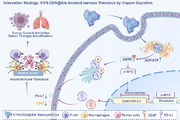

## 1 Introduction

Over the past few years, it has been demonstrated that blocking PD‐L1 alone to sensitize T‐cell‐mediated adaptive immunotherapy is not sufficient to counteract the multiple immune escape mechanisms of the tumor microenvironment [1, 2, 3]. It is necessary to combine with other immune checkpoint antagonistic molecules to enhance the benefits for patients, especially immune checkpoint antagonistic molecules that target innate immune cells [4, 5]. Numerous researchers believe that PD‐L1 and CD47 co‐regulation plays an essential role in avoiding tumor innate and adaptive immune evasion simultaneously [4, 6]. At present, CD47/PD‐L1 targeted bispecific antibodies like PF07257876, IBI‐322, and 6MW3211 have been widely studied in clinical trials to overcome innate and adaptive immune‐resistance at the same time [7, 8]. However, the excessive immune‐related adverse events caused by the on‐target off‐tumor immune‐toxicity of CD47/PD‐L1 targeted bispecific antibodies, especially red blood cell agglutination and dissolution, make the brutal reality that no CD47/PD‐L1 co‐regulation strategies have been proved yet in clinical settings [9, 10]. Thus, how to safely and effectively depress CD47 and PD‐L1 together is still a difficult problem in clinical.

To meet the energy demands of rapidly dividing tumor cells, a high copper status always exists in various types of cancers [11, 12]. With enough copper supply, the expression levels of mitochondrial electron transport chain cytochrome c oxidase (copper as a cofactor), indispensable for mitochondrial respiration, are raised, which then increases adenosine triphosphate (ATP) production [13, 14, 15, 16]. Due to the following decreased adenosine diphosphate (ADP)/ATP ratio, AMPK phosphorylation levels are reduced [17, 18, 19, 20]. Recently, it has been proven that AMPK pathway activation could inhibit c‐MYC protein expression through downregulating c‐MYC mRNA levels and accelerating c‐MYC destabilization and degradation by inducing the phosphorylation of c‐MYC at Thr400 [18, 21, 22]. Besides, c‐MYC suppression could decrease the expression levels of CD47 mRNA and protein in tumors via a transcriptional regulatory mechanism [23]. In consideration of the possible role of AMPK pathway in affecting PD‐L1 and CD47 expression, high copper status in tumors may be one of the vital causes that induce innate and adaptive immune resistance [23, 24, 25, 26, 27, 28]. Thus, we hypothesize that blocking copper trafficking in tumors may could be used as an efficient innate and adaptive immunotherapy sensitizing strategy.

**SCHEME 1:**
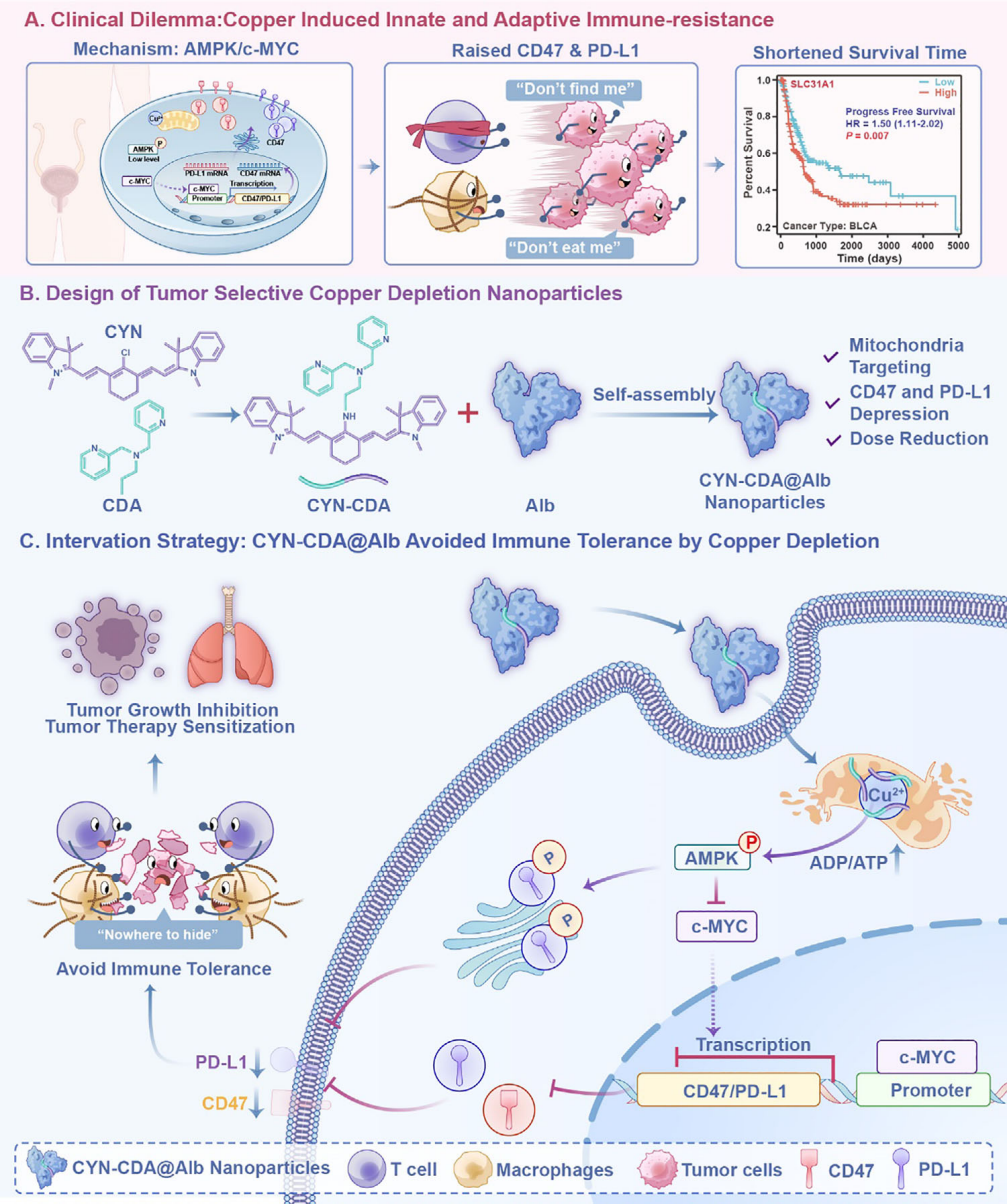
Schematic illustration of the relationship between copper and the innate and adaptive immune resistance. Copper would induce immunotherapy‐resistance with cold tumor characteristics by up‐regulation of “don't find me” signal PD‐L1 and “don't eat me” signal CD47. By tumor‐selective copper depletion via CYN‐CDA@Alb nanoparticles, immunotherapy sensitization with decreased PD‐L1 and CD47 expression was realized.

At present, some copper chelators like penicillamine, ammonium tetrathiomolybdate, and trientine are widely used in preclinical or clinical settings for copper‐related Wilson's disease [29, 30, 31]. Although these copper chelators could effectively deplete serum copper, the loss of their capacity to deplete tumor copper selectivity without affecting tissue or serum copper, as well as rapid elimination in vivo, makes them not that suitable for cancer‐specific copper depletion [32, 33, 34]. Here, we design tumor mitochondria‐targeting CYN‐CDA@Alb nanoparticles to deplete copper in the tumor with minimal side effects to healthy tissues. Specifically, CYN‐CDA@Alb nanoparticles are prepared by self‐assembly of the newly prepared mitochondrial‐targeting copper‐depleting molecule (CYN‐CDA) with albumin (Alb). As proved in this research, CYN‐CDA@Alb nanoparticles could decrease ATP generation to induce AMPK phosphorylation, which then depress the c‐MYC protein expression. Due to this, the expression of mRNA and protein levels of PD‐L1 (“don't find me” signal) and CD47 (“don't eat me” signal) is down‐regulated (Scheme 1). Following this, it is also revealed that CYN‐CDA@Alb, rather than some typical copper chelators, more effectively reversed innate and adaptive immune resistance by increasing T cell anti‐tumor activity and macrophage phagocytosis function. Moreover, CYN‐CDA@Alb alone or in combination therapy with radiotherapy increases the infiltration of T cells in tumors, inhibits tumor metastasis, and depresses local and abscopal tumor growth. All in all, the efficacy and safety of CYN‐CDA@Alb‐mediated copper depletion could be used as substitutes for CD47/PD‐L1 bispecific antibodies to potentiate cancer immunotherapy by awakening the innate and adaptive immune system.

## 2 Results

### 2.1 Copper Ions Upregulated PD‐L1 and CD47 to Suppress T Cell and Macrophage‐Mediated Immune Responses

SLC31A1 is a high‐affinity copper transporter localized primarily on the plasma membrane, responsible for mediating the influx of copper ions (Cu^2^^+^) from the extracellular to intracellular space [35]. Elevated expression of SLC31A1 is observed in multiple solid tumors, including bladder cancer and breast cancer (Figure S1A–C). As results revealed, immune‐related pathways, such as leukocyte migration and chemotaxis, were significantly enriched in the SLC31A1 low‐expression group, indicating a possible role of copper in modulating the tumor immune microenvironment (Figure S1D). By using single‐cell RNA sequencing (scRNA‐seq) re‐assay, it was found that high SLC31A1 expression was related to decreased T cell infiltration, impaired macrophage polarization, and limited B cell activity (Figures S1E–J, S2A,B). Next, we analyzed the correlation between SLC31A1 and the expression of PD‐L1 and CD47 across multiple cancer types, showing that the expression of SLC31A1 was positively correlated with that of PD‐L1 and CD47 in bladder cancer and breast cancer (Figure S1K, Figures S1 and S2). Thus, copper ions may affect PD‐L1 and CD47 expression in various tumors to induce immune resistance, which has not been clearly revealed yet.

To further validate these bioinformatic findings mentioned above, we conducted in vitro experiments using MB49, T24, and 4T1 cell lines to investigate whether high copper affects PD‐L1 and CD47 expression in vitro (Figures S1–S3). Western blot analyses revealed a dose‐dependent upregulation of PD‐L1 and CD47 protein levels in response to increasing concentrations of exogenous copper ions (Figures S1L–O, S3A–D). Functional assays further supported the immunosuppressive role of copper. In cytotoxicity assays, the presence of copper ions significantly impaired the ability of T cells to kill T24 tumor cells (Figures S1M, S3E). Additionally, flow cytometry analysis showed that copper ion treatment markedly suppressed the phagocytic activity of macrophages toward MB49 cells (Figure S1N). Finally, Kaplan–Meier survival analyses across multiple tumor types revealed that high SLC31A1 expression was significantly associated with poorer overall survival (Figure S1P), suggesting that copper accumulation mediated by SLC31A1 may promote immune evasion, accelerate tumor progression, and contribute to adverse clinical outcomes.

### 2.2 Copper Ions Regulated PD‐L1 and CD47 Expression via the AMPK/c‐MYC Signaling Axis

**FIGURE 1:**
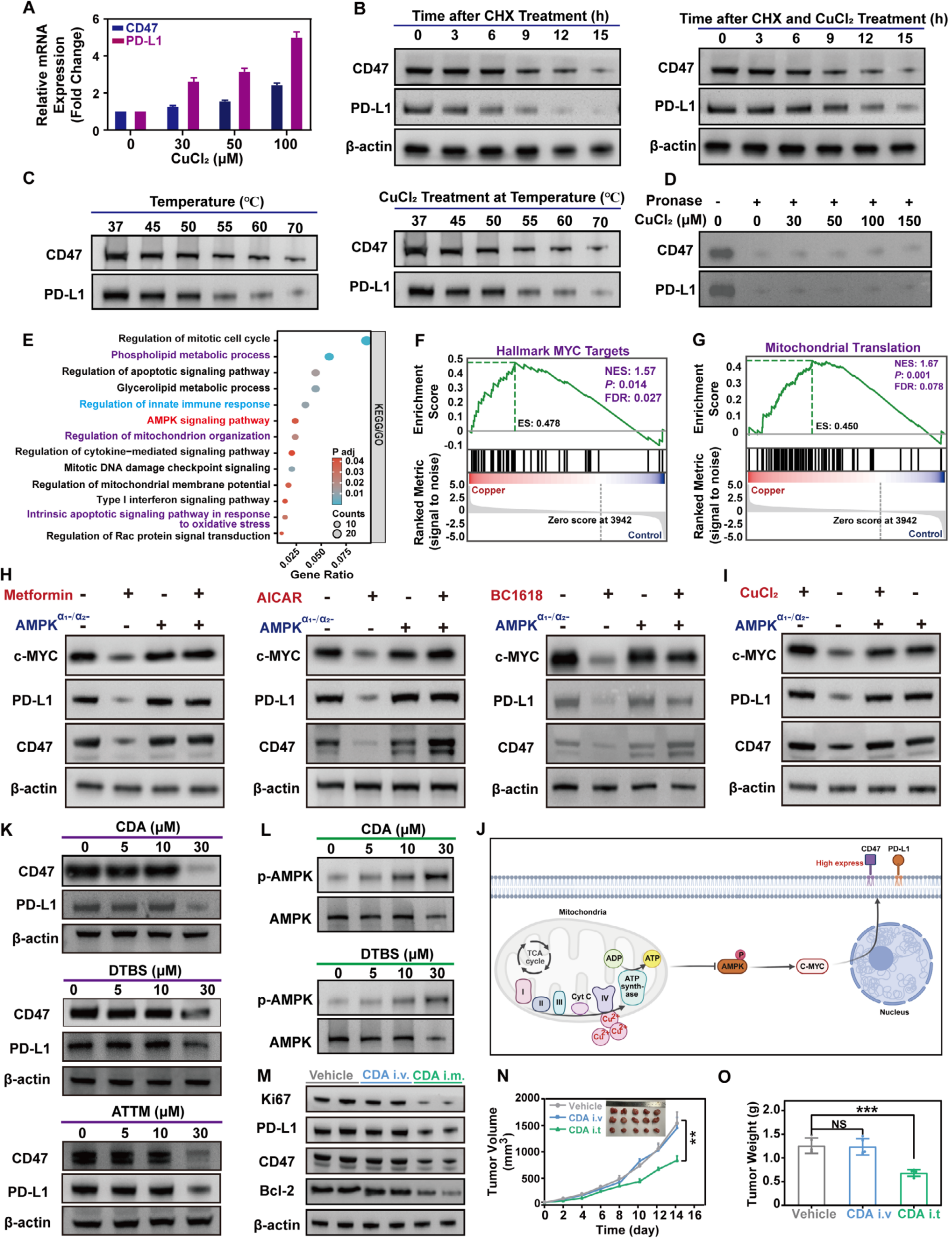
Copper ions regulated PD‐L1 and CD47 expression via the AMPK/c‐MYC axis. (A) Relative mRNA levels of PD‐L1 and CD47 assessed by RT‐qPCR in MB49 cells treated with increasing concentrations of CuCl_2_ (n = 3). (B) Protein half‐life assay showing the effect of CuCl_2_ treatment on the degradation kinetics of PD‐L1 and CD47 proteins in MB49 cells. (C,D) Evaluation of the interaction between CuCl_2_ and PD‐L1 or CD47 protein using CETSA and DART. (E–G) Proteomics profiling of CuCl_2_‐treated MB49 cells followed by KEGG, GO, and GSEA enrichment analysis of differentially expressed proteins. (H,I) Western blot analysis of c‐MYC, PD‐L1, and CD47 in MB49 cells transfected with shRNAs targeting AMPKα1 and AMPKα2, in the presence or absence of AMPK inhibitors and CuCl_2_. (J) Schematic model of CuCl_2_‐mediated regulation of PD‐L1 and CD47 expression via the AMPK/c‐MYC signaling axis. (K,L) Western blotting analysis of PD‐L1, CD47, p‐AMPK, and total AMPK expression in MB49 cells cultured in copper‐enriched medium for 8 h, after which were treated with various copper chelators. (M) Western blot analysis of Ki67, PD‐L1, CD47, and Bcl‐2 in tumor tissues from mice receiving either intratumoral (i.t) or intravenous (i.v) injections of CDA. (N) Representative tumor images and tumor volume growth curves of CDA‐treated MB49 tumor‐bearing mice (n = 5). (O) Tumor weight measurements at the study endpoint in the same treatment groups (n = 5). Statistical analysis was performed via a two‐tail Student's *t*‐test. ^*^ *p* < 0.05, ^**^ *p* < 0.01, ^***^ *p* < 0.001, NS *p* > 0.05.

To our best knowledge, the mechanism by which copper ions affect the expression of immune checkpoint molecules PD‐L1 and CD47 had not been proven yet [36, 37, 38]. As the RT‐PCR results indicated, copper ions not only elevated the protein expression of PD‐L1 and CD47 but also significantly upregulated their mRNA levels, suggesting that the regulatory effect occurs, at least in part, at the transcriptional level (Figure 1A). In addition, protein half‐life assays indicated that copper ions extended the half‐life of PD‐L1, showing that copper ions inhibited PD‐L1 degradation (Figure 1B). Moreover, as the results of Cellular Thermal Shift Assay (CETSA) and Drug Affinity Responsive Target Stability (DARTS) assays showed, copper ions may not affect PD‐L1 and CD47 expression through direct identification and combination (Figure 1C,D). To systematically explore the potential mechanisms underlying copper‐induced upregulation of PD‐L1 and CD47, we conducted proteomic profiling of MB49 cells treated with copper ions (Figure 1E,G). GO, KEGG, and GSEA analyses revealed significant alterations in mitochondrial function and a marked suppression of AMPK signaling following copper treatment (Figure 1E,G). As a central energy sensor, AMPK plays a vital role in cellular metabolic homeostasis and has been implicated in tumor immunity [21, 24]. Previous studies have shown that phosphorylated AMPK (p‐AMPK) can promote the ubiquitination and degradation of PD‐L1 by phosphorylating it in the endoplasmic reticulum, thereby negatively regulating PD‐L1 expression [25, 26]. Copper is an essential cofactor in mitochondrial function, particularly for cytochrome c oxidase (COX, complex IV of the electron transport chain), which could affect ATP generation to depress AMPK pathway activation [33, 37]. Thus, copper ions may affect PD‐L1 and CD47 expression by affecting the AMPK pathway.

To confirm the inhibitory effect of copper on AMPK signaling, we modestly elevated intracellular copper levels in MB49, T24, and 4T1 cells. Western blot analysis revealed consistent suppression of AMPK phosphorylation across all three cell lines, further confirming the negative regulatory role of copper on AMPK activity (Figure S3F–H). Moreover, GSEA results suggested that copper ions significantly activate pathways associated with the oncogene c‐MYC (Figure 1F,G, Figure S4). Previous studies have identified c‐MYC as a transcriptional enhancer of both PD‐L1 and CD47 [23]. Based on these findings, we hypothesized that copper may upregulate PD‐L1 and CD47 by inhibiting AMPK, thereby relieving suppression of c‐MYC activity [23]. To validate this hypothesis, we generated a stable AMPK knockdown MB49 cell line and treated cells with CuCl_2_ alone or in combination with AMPK agonists (Figure 1H,I, Figure S5). Western blot analysis showed that in AMPK‐deficient cells, c‐MYC, PD‐L1, and CD47 levels remained elevated regardless of CuCl_2_ treatment, whereas in wild‐type cells, AMPK activation or the absence of CuCl_2_ partially reversed this upregulation (Figure 1H,I, Figure S5). Taken together, these findings demonstrated that copper ions promoted the expression of PD‐L1 and CD47 by inhibiting AMPK and activating downstream c‐MYC, thereby establishing the critical role of the copper/AMPK/c‐MYC axis in mediating tumor immune evasion.

### 2.3 Copper Chelators Reversed Copper‐Induced Tumor Immune Evasion In Vitro and In Vivo

Copper chelators are compounds that selectively bind and remove free copper ions from the body, which are traditionally used for treating disorders associated with copper overload, such as Wilson's disease [14, 39]. These agents exert antitumor effects through multiple mechanisms, including the inhibition of copper‐dependent signaling pathways, disruption of copper‐containing enzyme functions, and impairment of mitochondrial metabolism [40]. Given the pivotal role of copper ions in mediating tumor immune evasion, we hypothesized that copper chelators could restore antitumor immunity by reversing copper‐induced immunosuppressive effects (Figure 1). To test this, MB49 cells were first exposed to a copper‐enriched environment to simulate a high‐copper tumor microenvironment [33]. Subsequently, cells were then treated with three representative copper chelators: CDA (Tris(2‐pyridylmethyl)amine), DTBS (Bis(thiobenzoyl)disulfide), and ATTM (Ammonium Tetrathiomolybdate) (Figure 1K). Western blot analysis revealed that copper exposure significantly induced the expression of PD‐L1 and CD47, whereas treatment with copper chelators led to a dose‐dependent downregulation of both proteins (Figure 1K; Figure S6A–C). Additionally, all three chelators were capable of activating the energy sensor AMPK pathway (Figure 1K; Figure S6D–G). To further validate these findings in vivo, we established a syngeneic MB49 tumor‐bearing mouse model and administered intratumoral injections of CDA (Figure 1M–O). The treatment significantly inhibited tumor growth compared to controls with decreased PD‐L1 and CD47 expression (Figure 1M–O; Figure S6H). Based on our in vivo findings, intratumoral administration of CDA exhibited superior antitumor efficacy compared to intravenous injection, suggesting that local delivery strategies could enhance therapeutic outcomes (Figure 1O; Figure S6I). To sum up, these results indicated that copper chelators reversed copper‐induced tumor immune evasion in vitro and in vivo by depressing PD‐L1 and CD47 expression.

### 2.4 Synthesis and Characterization of CYN‐CDA@Alb Nanoparticles

**FIGURE 2:**
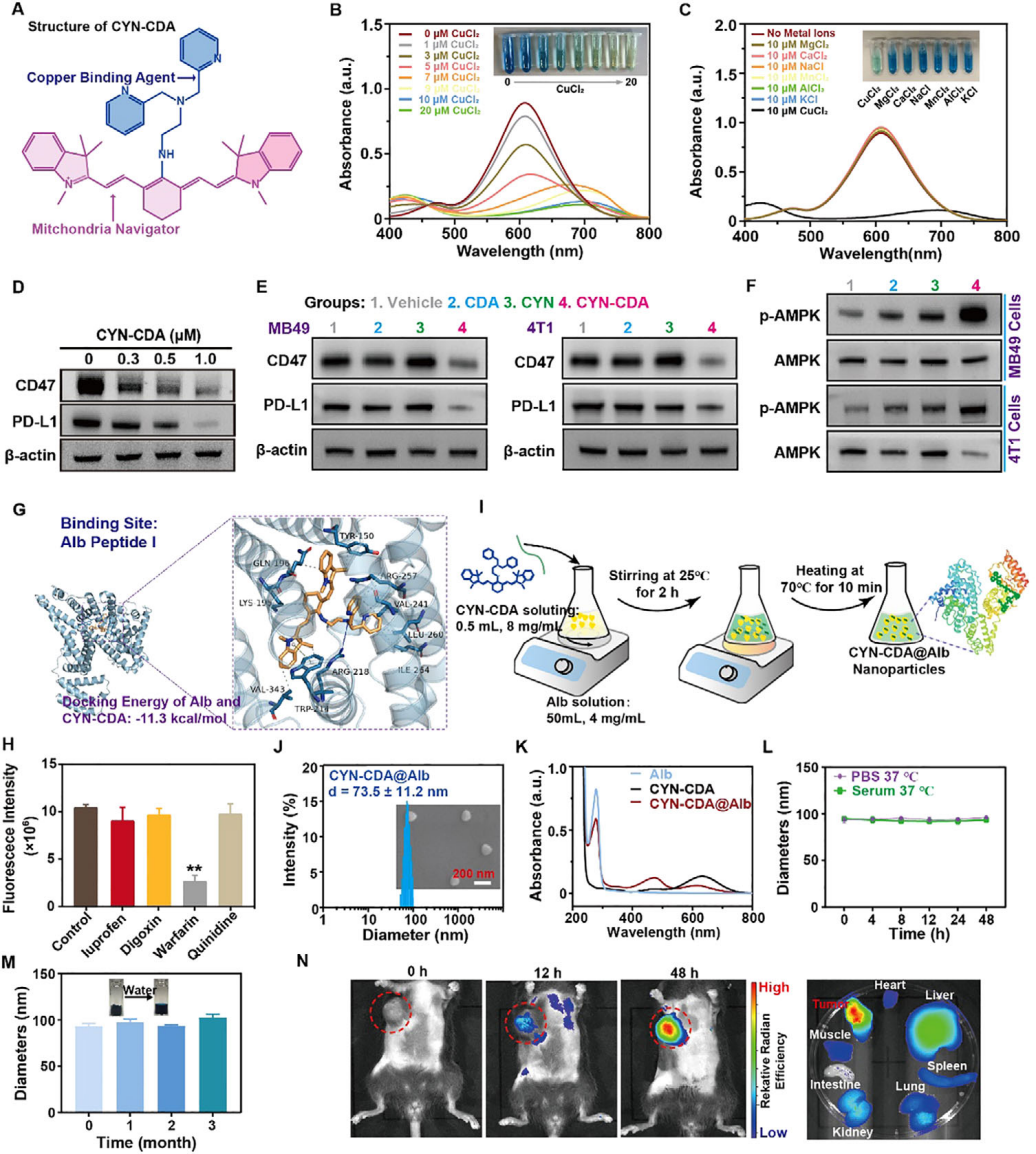
Preparation and characterization of CYN‐CDA@Alb nanoparticles. (A) Schematic illustration of CYN‐CDA structure. (B) UV absorbance curve of CYN‐CDA (10 µm) after incubation with various concentrations of CuCl_2_. (C) UV absorbance curve of CYN‐CDA (10 µm) after incubation with 10 µm concentration of different metal ions (Cu^2+^, Na^+^, Mg^2^^+^, K^+^, Ca^2^^+^; Zn^2^^+^, Mn^2^^+^). (D) Detection of the expression levels of CD47 and PD‐L1 in MB49 cells by western blot assay after treatment with different concentrations of CYN‐CDA. (E,F) Detection of the expression levels of CD47, pAMPK, AMPK, and PD‐L1 protein in MB49 cells by western blot assay after different treatments. (G) Molecular docking model of CYN‐CDA with Alb. (H) Competitive binding assays using site‐specific inhibitors (warfarin, ibuprofen, digoxin, quinidine targeting albumin sites I, II, III, and α_1_‐acid glycoprotein) to determine CYN‐CDA binding site on Alb. (I) Preparation process of CYN‐CDA@Alb nanoparticles. (J) Scanning electron microscopy (SEM) image and dynamic light scattering (DLS) measurement of CYN‐CDA@Alb, scale bar = 200 nm. (K) UV–vis absorption spectrum of CYN‐CDA@Alb in deionized water. (L,M) Stability assessment of CYN‐CDA@Alb over time and under different storage conditions (n = 3). (N) In vivo real‐time near‐infrared (NIR) fluorescence imaging of MB49 tumor‐bearing mice after intravenous injection of CYN‐CDA@Alb (CYN‐CDA: 2 mg/kg). Representative NIR fluorescence images of tumors and normal organs collected at 48 h post‐treatment.

At present, the major limitation of copper chelators in clinical applications is the requirement for high doses to achieve therapeutic effects, which can lead to significant adverse effects [14]. To further improve the efficacy and tumor targeting specificity of the copper chelation strategy, we designed a structurally modified mitochondria‐targeting copper depletion molecule (CYN‐CDA) by introducing a tumor‐mitochondria targeting moiety CYN to the copper depletion agent CDA (Figure 2A; Figure S7). UV–vis spectroscopy analysis confirmed the copper‐binding capacity of CYN‐CDA, demonstrating a high degree of selectivity for copper ions over other metal ions (Figure 2B,C). Functionally, MB49 cells treated with CYN‐CDA showed significant downregulation of PD‐L1 and CD47 expression at concentrations as low as 1 µM via inducing AMPK phosphorylation, while CDA required 30 µm to achieve comparable effects (Figure 2D–F; Figure S8A,B). These results suggested that CYN modification enhanced the immunomodulatory potency of CDA and potentially reduced its dosage‐related toxicity (Figure 2D–F). To further improve biocompatibility and tumor targeting, we developed an Alb‐based nanoformulation, CYN‐CDA@Alb. Molecular docking simulations indicated a stable binding affinity between CYN‐CDA and Alb (Figure 2G). Additionally, fluorescence quenching assays confirmed the specific interaction of CYN‐CDA with Alb binding site I, as evidenced by competitive displacement with site‐specific ligands such as quinidine, ibuprofen, digoxin, and warfarin, resulting in significant fluorescence reduction (Figure 2H). The CYN‐CDA@Alb nanoparticles were prepared as outlined (Figure 2I), and their size was characterized by scanning electron microscopy (SEM) and dynamic light scattering (DLS), showing a typical nanoparticle morphology with an average diameter of 73.5 ± 11.2 nm (Figure 2J). UV–vis absorption spectra revealed a characteristic peak at approximately 620 nm, confirming successful encapsulation of CYN‐CDA (Figure 2K). Particle size remained stable under various storage conditions, indicating desirable nanoparticle stability (Figure 2L,M). These findings suggested that CYN‐CDA@Alb not only retained copper‐chelating capability but also elicited potent immune‐regulatory effects, thereby enhancing antitumor immunity within the tumor microenvironment.

Considering the proven fact that Alb nanoparticles could accumulate in tumors by passive and active targeting, we believed that CYN‐CDA@Alb may could selectively accumulate in tumors (Figure 2N) [41, 42]. As results indicated, in vivo fluorescence imaging confirmed the specific enrichment of CYN‐CDA@Alb at tumor sites (Figure 2N). Then, to evaluate the in vivo safety profile of CYN‐CDA@Alb, we performed a hemolysis assay to assess its effect on murine red blood cells, showing that CYN‐CDA@Alb did not induce significant hemolysis (Figure S9A,B). Furthermore, no significant differences in creatinine (CRE), blood urea nitrogen (BUN), aspartate aminotransferase (AST), and alanine aminotransferase (ALT) levels were observed between the treatment and control groups (Figure S9C–F). Moreover, no apparent pathological alterations were observed in these tissues following CYN‐CDA@Alb treatment compared to controls, suggesting an absence of organ‐level toxicity (Figure S9G). Collectively, these results demonstrated that CYN‐CDA@Alb possessed favorable biocompatibility, tumor‐targeting capacity, and biosafety in vivo.

### 2.5 CYN‐CDA@Alb Suppressed Tumor Progression and Enhanced Antitumor Immune Responses In Vitro

**FIGURE 3:**
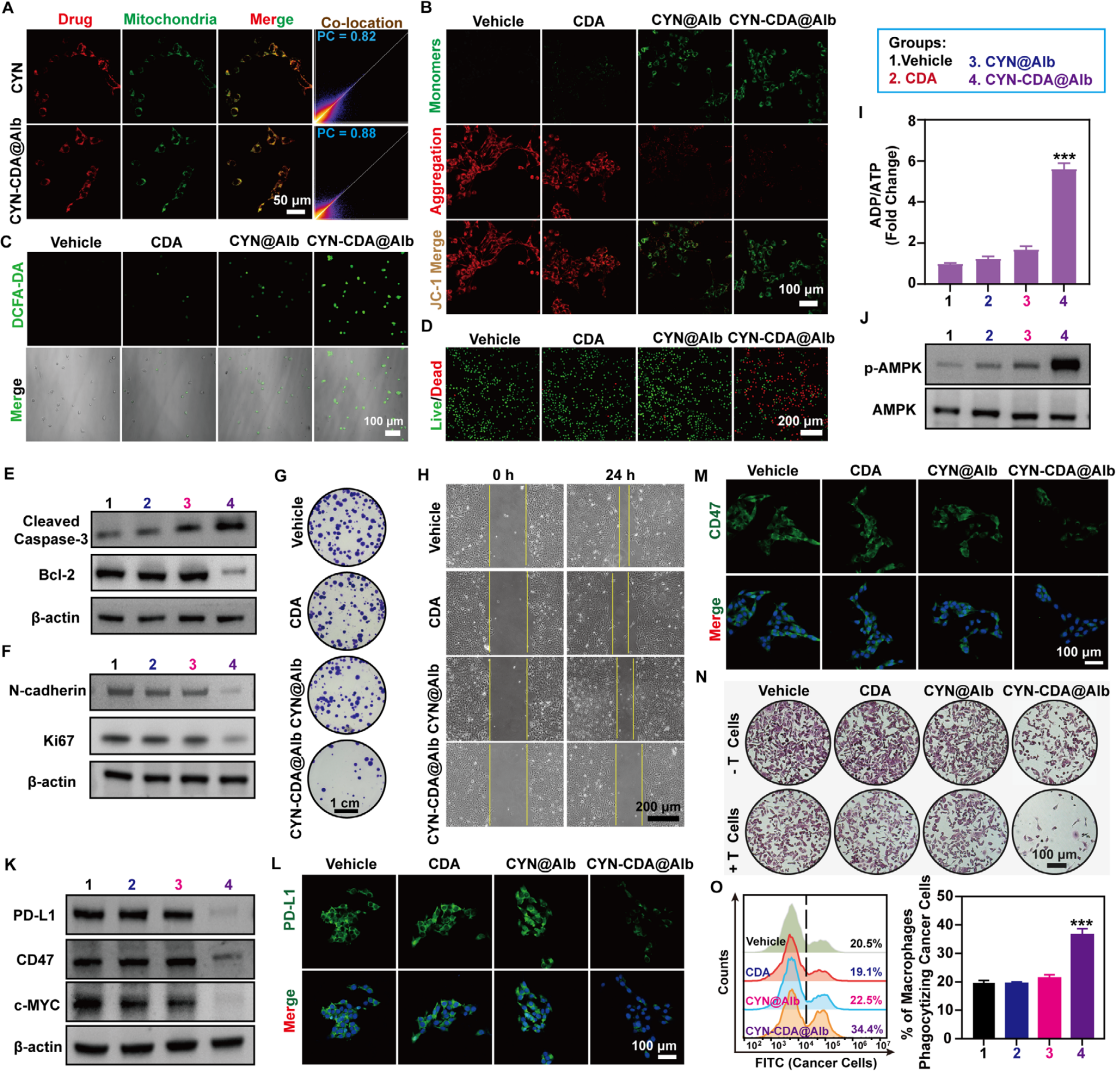
Antitumor effects and mechanism of CYN‐CDA@Alb in vitro. (A) Co‐localization of CYN‐CDA@Alb nanoparticles with mitochondria in MB49 cells visualized by confocal microscopy using MitoTracker Green, scale bar = 50 µm. (B) Detection of the mitochondrial membrane potential (ΔΨm) by JC‐1 staining in MB49 cells treated with different drugs (2 µm for all groups), scale bar = 100 µm. (C) Evaluation of the intracellular ROS levels by using DCFH‐DA probe in various drug‐treated groups, scale bar = 200 µm. (D) Live/dead cell staining to evaluate cytotoxicity of different treatments in MB49 cells, scale bar = 200 µm. (E,F) Western blot analysis of Cleaved Caspase‐3, Bcl‐2, N‐cadherin, and Ki67 following drug treatment. (G) Colony formation assay assessing the proliferation ability of MB49 cells under different treatment conditions. (H) Wound healing assay to evaluate the migration capability of MB49 cells after drug treatment. (I) Measurement of the ADP/ATP ratio in MB49 cells at 12 h after treatment with various drugs. (J) Western blot analysis of p‐AMPK and AMPK following drug treatments. (K) Western blot analysis of PD‐L1, CD47, and c‐MYC protein levels in MB49 cells treated with different drugs. (L,M) CLSM images showing PD‐L1 and CD47 immunofluorescence in MB49 cells across different treatment groups, scale bar = 100 µm. (N) Evaluation of cytotoxic T cell‐mediated killing of MB49 cells pretreated with different drugs after co‐culture with activated T cells, scale bar = 100 µm. (O) Flow cytometric analysis of phagocytosis of MB49 cells by macrophage cells under various treatments. Statistical analysis was performed via a two‐tail Student's *t*‐test. ^*^ *p* < 0.05, ^**^ *p* < 0.01, ^***^ *p* < 0.001.

As previously, heptamethine cyanine molecule‐loaded alb nanoparticles possessed the desired tumor‐selective accumulation behavior [19, 43, 44]. To evaluate whether CYN‐CDA@Alb exhibited similar characteristic, we detected the accumulation behavior of CYN‐CDA@Alb in different cells, during which we found that more CYN‐CDA@Alb was distributed in tumors rather than normal cells (Figure S10A,B). To further evaluate whether CYN‐CDA@Alb exhibited ideal mitochondrial targeting capacity like CYN, the co‐localization of CYN‐CDA@Alb with mitochondrial markers in tumor cells was investigated (Figure 3A,B). As results indicated, CYN‐CDA@Alb showed typical mitochondrial targeting capability, which then resulted in the disruption of the mitochondrial membrane potential (ΔΨm), copper disruption, and mitochondrial dysfunction with decreased oxygen consumption (Figure 3A,B; Figure S10C,D). Mitochondrial impairment was then accompanied by excessive accumulation of intracellular reactive oxygen species (ROS) after CYN‐CDA@Alb treatment, as evidenced by enhanced DCFH‐DA fluorescence (Figure 3C). Consequently, tumor cell apoptosis was significantly increased and tumor cell growth was inhibited with increased expression of Cleaved Caspase‐3 and decreased expression of the anti‐apoptotic protein Bcl‐2 (Figure 3E–G). Apart from this, the epithelial‐mesenchymal transition (EMT) detected by wound healing assays demonstrated that CYN‐CDA@Alb significantly suppressed the migration of MB49 tumor cells via decreased expression of N‐cadherin (Figure 3F,G; Figure S8C–E). We next evaluated the cytotoxicity of CDA, CYN@Alb, and CYN‐CDA@Alb in MB49, T24, and 4T1 cells using CCK‐8 assays and calculated their IC50 values (Figure S8F–H). CYN‐CDA@Alb displayed robust anti‐proliferative activity across multiple tumor cell lines. Thus, CYN‐CDA@Alb more effectively inhibited tumor cell growth, induced tumor cell apoptosis, and slowed tumor metastasis in vitro.

Apart from the findings mentioned above, it was also revealed that ADP/ATP ratio was raised by CYN‐CDA@Alb to induce the phosphorylation of AMPK protein (Figure 3I–L; Figure S8I). Similar to CYN‐CDA, CYN‐CDA@Alb then obviously inhibited PD‐L1 and CD47 expression via the AMPK/c‐MYC axis (Figure 3L). These results suggested the possible capacity of CYN‐CDA@Alb in reversing the immunosuppressive tumor microenvironment (Figure 3L,M; Figure S8J,K). Functionally, T cell cytotoxicity assays revealed that CYN‐CDA@Alb enhanced the killing capacity of T cells against tumor cells (Figure 3N; Figure S11). In addition, flow cytometry analysis showed that CYN‐CDA@Alb increased the phagocytic activity of macrophages toward tumor cells (Figure 3O). Moreover, pre‐treatment CYN‐CDA@Alb with T cells and macrophage cells had little influence on the cell viability of T cells and macrophage cells (Figure S11). Collectively, these findings demonstrate that CYN‐CDA@Alb not only inhibited malignant tumor progression but also elicited robust activation of both innate and adaptive antitumor immune responses.

### 2.6 CYN‐CDA@Alb Promoted Antitumor Immunity and Prolonged Mice Survival In Vivo

**FIGURE 4:**
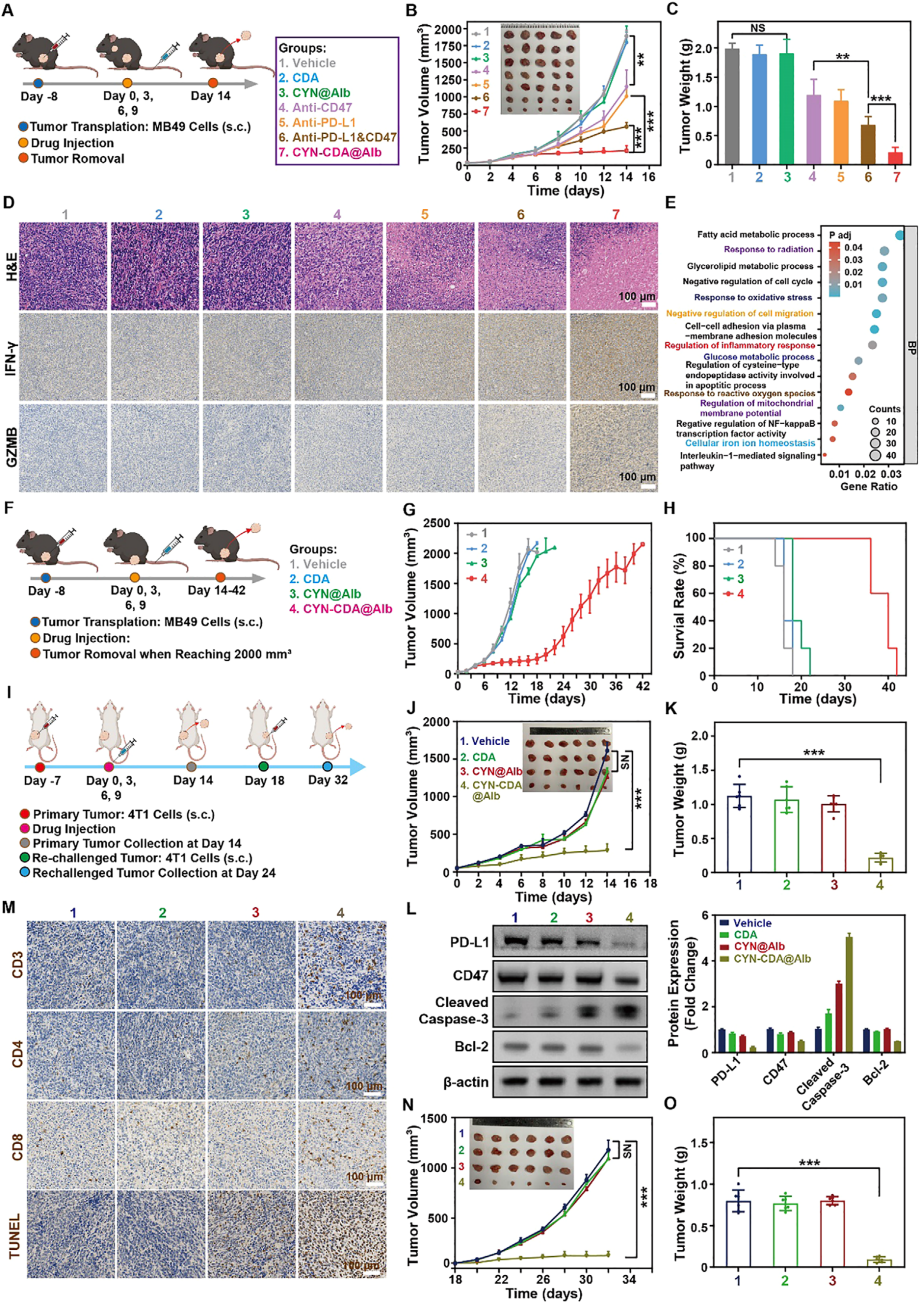
Copper‐chelating nanoparticles CYN‐CDA@Alb boosted anti‐tumor immunity and long‐term immune memory in vivo. (A) Schematic illustration of the mouse anti‐tumor immunotherapy model. (B) MB49 tumor images and MB49 tumor volume growth curves of mice under different treatments (n = 5). (C) Tumor weight statistics of the anti‐tumor immunotherapy model (n = 5). (D) Representative H&E staining and immunohistochemistry of IFN‐γ and GZMB in tumor sections from different treatment groups, scale bar = 100 µm. (E) GO and KEGG enrichment analysis of tumor proteomics from Vehicle and CYN‐CDA@Alb‐treated groups. (F) Schematic diagram of the MB49 tumor‐bearing mouse survival model following drug treatment. (G) Tumor volume curves of mice in the survival model under different treatments (n = 5). (H) Kaplan‐Meier survival analysis of the mouse survival model (n = 5). (I) Schematic illustration of the immune memory model in 4T1 tumor‐bearing mice. (J) Representative tumor images and tumor volume curves of primary 4T1 tumors in the immune memory model (n = 6). (K) Tumor weight statistics of primary 4T1 tumors in the immune memory model (n = 6). (L) Quantification and western blot analysis of PD‐L1, CD47, Cleaved Caspase‐3, and Bcl‐2 protein in primary 4T1 tumors (n = 3). (M) Representative immunohistochemistry staining of CD3, CD4, CD8, and TUNEL staining of primary tumors in the immune memory model. (N) Tumor images and volume curves of distant 4T1 tumors in the immune memory model (n = 6). (O) Tumor weight statistics of distant 4T1 tumors in the immune memory model (n = 6). Statistical analysis was performed via a two‐tail Student's *t*‐test. ^*^ *p* < 0.05, ^**^ *p* < 0.01, ^***^ *p* < 0.001, NS *p* > 0.05.

Given the previously demonstrated in vitro anti‐tumor growth and immune‐enhancing effects of CYN‐CDA@Alb, we further evaluated its immunotherapeutic efficacy in the MB49 murine bladder cancer model (Figure 4A). Results showed that treatment with CYN‐CDA@Alb, as well as three antibody‐based regimens (anti‐PD‐L1, anti‐CD47, and their combination), effectively inhibited tumor growth (Figure 4B,C; Figures S12,S13). Interestingly enough, based on tumor volume measurements, hematoxylin and eosin (H&E) staining, and IFN‐γ immunohistochemistry, the CYN‐CDA@Alb group exhibited the most pronounced effects in terms of tumor suppression, induction of tumor cell apoptosis, and immune activation (Figure 4D). To further elucidate the underlying mechanisms, proteomic profiling was performed on tumor tissues from the Vehicle and CYN‐CDA@Alb groups, followed by GO/KEGG and GSEA enrichment analyses. As results indicated, CYN‐CDA@Alb significantly activated multiple pathways related to immune responses and tumor apoptosis (Figure 4E; Figure S12). Flow cytometric analysis of tumor‐infiltrating lymphocytes revealed that the proportions of CD3^+^, CD4^+^, and CD8^+^ T cells were markedly increased in CYN‐CDA@Alb group compared to other treatment groups, further confirming its strong ability to elicit antitumor immune responses in vivo (Figure S14). In a survival study, using a tumor volume of 2000 mm^3^ as the endpoint, we established a survival model in MB49 tumor‐bearing mice treated with CYN‐CDA@Alb. As results demonstrated, CYN‐CDA@Alb significantly prolonged mice survival (Figure 4F–H; Figure S15A), indicating its promising potential for improving survival outcomes and clinical prognosis.

Tumor recurrence and metastasis remain major challenges in current clinical oncology [18]. To investigate the potential of CYN‐CDA@Alb in preventing tumor relapse and inhibiting distant metastasis, we established both a primary and recurrent tumor model using 4T1 murine breast cancer cells, as well as a 4T1 lung metastasis model (Figure 4I). In the primary tumor model, CYN‐CDA@Alb treatment significantly inhibited tumor growth (Figure 4J,K; Figure S15B). Western blot analysis of tumor tissues further demonstrated that CYN‐CDA@Alb downregulated the expression of immunosuppressive molecules PD‐L1 and CD47, while upregulating cleaved caspase‐3 and downregulating Bcl‐2, suggesting enhanced activation of both innate and adaptive immunity and effective induction of tumor cell apoptosis (Figure 4L). Immunohistochemical staining analysis revealed a marked increase in intratumoral infiltration of CD3^+^, CD4^+^, and CD8^+^ T cells following treatment, indicating robust immune activation (Figure 4M). In the recurrence model, CYN‐CDA@Alb also significantly suppressed postoperative tumor regrowth, suggesting its potential to reduce recurrence risk, possibly through the induction of immunological memory (Figure 4N,O; Figures S15C, S16A). Moreover, based on our previous in vitro findings that CYN‐CDA@Alb may inhibit tumor cell migration, we performed a transwell invasion assay. Results confirmed that CYN‐CDA@Alb significantly impaired the invasive capacity of tumor cells (Figure S16B,C). To further validate its anti‐metastatic potential in vivo, we employed a 4T1 lung metastasis model (Figure S16D). Quantification of metastatic nodules and H&E staining of lung tissues demonstrated that CYN‐CDA@Alb effectively reduced pulmonary metastases, providing further evidence of its anti‐metastatic efficacy (Figure S16E–G). All in all, CYN‐CDA@Alb could reverse immune‐resistance by depressing PD‐L1 and CD47 to inhibit local tumor growth, prevent tumor recurrence, and suppress tumor metastasis.

### 2.7 CYN‐CDA@Alb Synergizes with Radiotherapy to Suppress both Primary and Distant Tumor Growth and Prolong Survival

**FIGURE 5:**
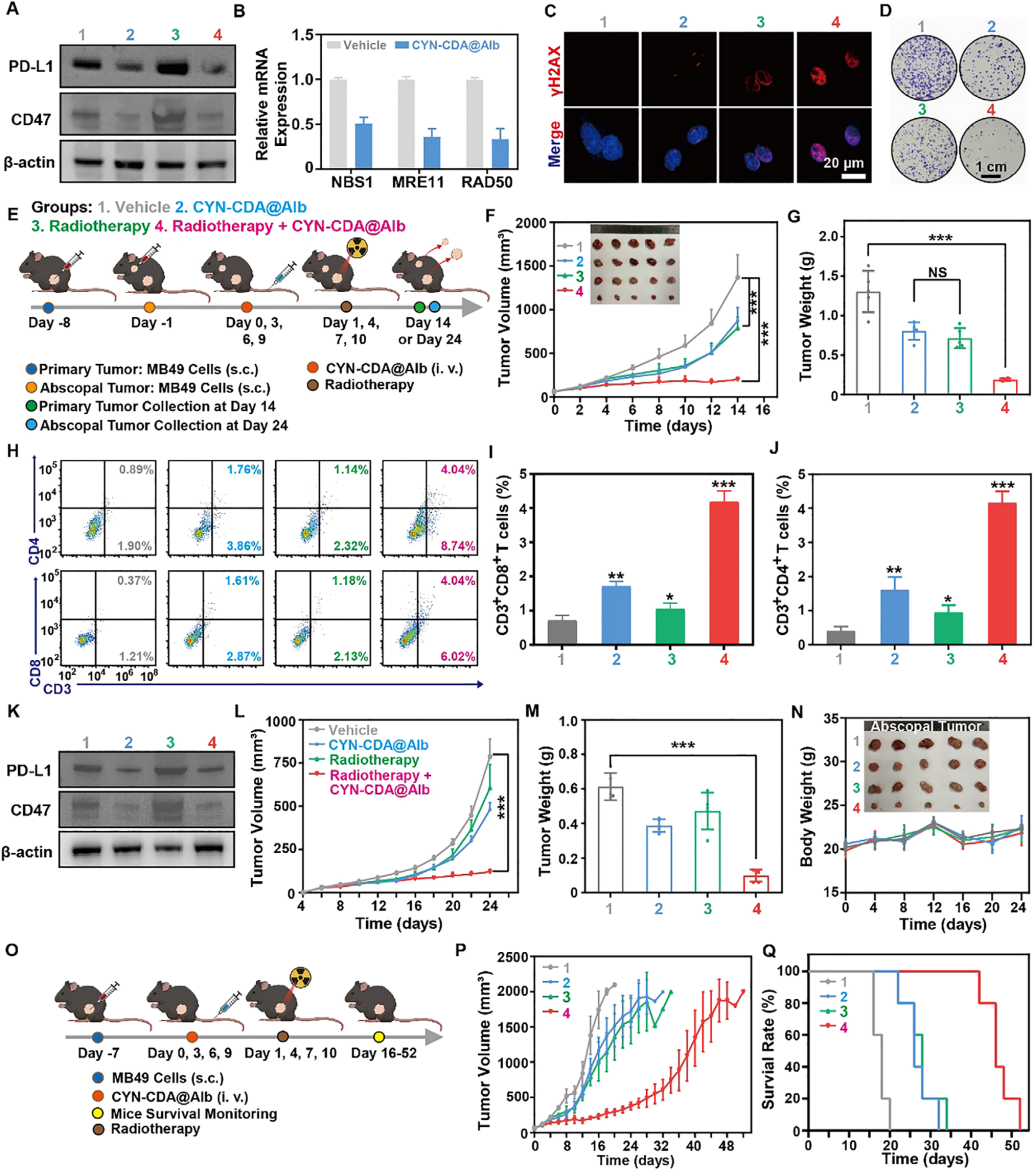
CYN‐CDA@Alb synergized with radiotherapy to enhance immune activation and improve antitumor efficacy in vitro and in vivo. (A) Western blot analysis of PD‐L1 and CD47 expression in MB49 cells treated with PBS, CYN‐CDA@Alb, radiotherapy (3 Gy), or the combination of CYN‐CDA@Alb and radiotherapy. (B) RT‐qPCR analysis of DNA damage repair genes (NBS1, MRE11, and RAD50) after CYN‐CDA@Alb treatment. (C) Representative CLSM images of γH2AX in MB49 cells, indicating DNA damage after various treatments. (D) Colony formation assay of MB49 cells following different treatments. (E) Schematic diagram of in situ tumor mouse models used for evaluating CYN‐CDA@Alb‐mediated radiotherapy enhancement. (F) Tumor images and tumor growth curves of in situ tumors in the radiotherapy‐sensitization model (n = 5). (G) Tumor weight statistics of in situ tumors (n = 5). (H–J) Flow cytometry analysis and quantification of CD3^+^, CD4^+^, and CD8^+^ T cells infiltrating in situ tumors from different treatment groups. (K) Western blot analysis of PD‐L1 and CD47 expression in tumors collected from each treatment group. (L) Growth curves of abscopal MB49 tumors (n = 5). (M) Tumor weight analysis of abscopal MB49 tumors (n = 5). (N) Tumor images and growth curves of abscopal tumors (n = 5). (O) Schematic of survival model evaluating combination therapy. (P) Tumor growth curves under different treatment conditions in the survival model (n = 5). (Q) Kaplan–Meier survival analysis of mice in the radiotherapy‐enhancement survival model. Statistical analysis was performed via a two‐tail Student's *t*‐test. ^*^ *p* < 0.05, ^**^ *p* < 0.01, ^***^ *p* < 0.001.

Previous studies had shown that radiotherapy can upregulate the expression of immunosuppressive molecules such as PD‐L1 and CD47 in tumor cells, thereby contributing to the establishment of a radiotherapy‐induced immunosuppressive microenvironment [45, 46]. Based on this, we hypothesized that CYN‐CDA@Alb might enhance antitumor efficacy when combined with radiotherapy (Figure 5). To prove this, we first investigated the synergistic mechanisms between radiotherapy and CYN‐CDA@Alb in vitro. Western blot analysis revealed that CYN‐CDA@Alb effectively inhibited radiation‐induced upregulation of PD‐L1 and CD47 protein (Figure 5A; Figure S17A). RT‐qPCR analysis further confirmed that the transcription levels of DNA damage repair‐related genes (NBS1, MRE11, and RAD50) were significantly reduced upon CYN‐CDA@Alb treatment (Figure 5B) [47, 48]. Confocal microscopy showed elevated expression of γ‐H2AX in tumor cells treated with the combination therapy, indicating increased DNA damage and a potential radio‐sensitizing effect of CYN‐CDA@Alb (Figure 5C). Consistently, clonogenic assays demonstrated stronger tumor cell killing in the combination group compared to monotherapies (Figure 5D; Figure S17B).

Encouraged by the in vitro findings, we next evaluated the in vivo efficacy of the combination treatment using a bilateral MB49 tumor model, where only the primary (proximal) tumor received irradiation (Figure 5E). As results indicated, combination therapy of CYN‐CDA@Alb and radiotherapy more significantly suppressed the growth of irradiated primary tumors when compared with the radiotherapy group, at least partly owing to the capacity of CYN‐CDA@Alb in avoiding the possibly raised PD‐L1 and CD47 expression induced by radiotherapy (Figure 5F–K; Figure S17C). Then, flow cytometric analysis of proximal tumors revealed significantly increased infiltration of CD3^+^, CD4^+^, and CD8^+^ T cells in the combination group, indicating a critical role of T cell‐mediated immune responses in the synergistic effect (Figure 5G–I; Figure S17D). More interestingly, combination therapy of CYN‐CDA@Alb and radiotherapy also inhibited the progression of non‐irradiated distant tumors, suggesting the induction of a systemic antitumor immune response (abscopal effect) (Figure 5L–N). At last, we assessed the impact of combination therapy on mouse survival using the MB49 tumor model (Figure 5O–Q; Figure S18). With tumor volume reaching 2000 mm^3^ as the study endpoint, results showed that CYN‐CDA@Alb combined with radiotherapy significantly prolonged overall survival, further supporting its potential to enhance the radiotherapeutic efficacy and highlighting its promising translational potential.

### 2.8 Clinical Data Further Reveal Immune Evasion Status in Tumors under High‐Copper Conditions

**FIGURE 6:**
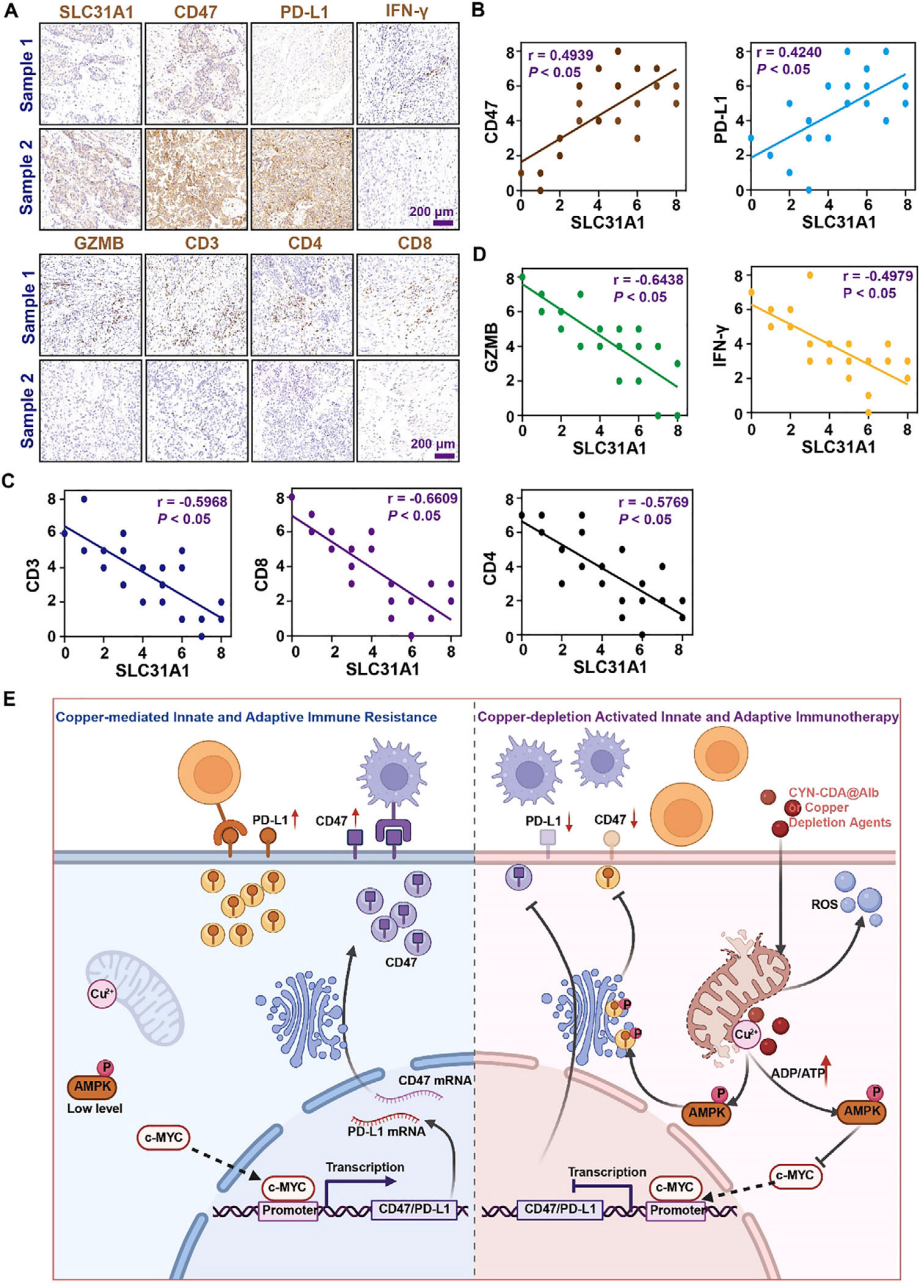
Clinical relevance and mechanism of CYN‐CDA@Alb‐induced anti‐tumor immune sensitization. (A) Representative immunohistochemistry staining images of tumor tissues from clinical bladder cancer patients, showing expression levels of SLC31A1, CD47, PD‐L1, IFN‐γ, GZMB, CD3, CD4, and CD8. (B‐D) Correlation analysis between SLC31A1 and immune markers, including CD47, PD‐L1, CD3, CD4, CD8, GZMB, and IFN‐γ in patient tissues. (E) Proposed schematic illustration of the mechanism by which CYN‐CDA@Alb or copper depletion agents regulate tumor immunity to enhance anti‐tumor immune responses.

To further validate the pivotal role of copper ions in tumor immune regulation and assess their potential clinical relevance, we collected tumor tissue samples from patients with bladder cancer (Figure 6). The expression levels of immune‐related proteins, including SLC31A1, PD‐L1, CD47, IFN‐γ, GZMB, CD3, CD4, and CD8, were analyzed by immunohistochemistry (Figure 6A). Based on the percentage of positive cells and staining intensity, immunohistochemical staining results were quantitatively evaluated and visualized in scatter plots (Figure 6B–D) [49]. The analysis revealed that SLC31A1 expression was significantly positively correlated with PD‐L1 and CD47, but negatively correlated with IFN‐γ, GZMB, CD3, CD4, and CD8 (Figure 6B–D). These findings suggested that elevated SLC31A1 expression in bladder cancer tissues may be associated with increased levels of immunosuppressive molecules and decreased effector T cell activity, leading to an enhanced immunosuppressive tumor microenvironment and potentially poorer immune responses and clinical outcomes. This clinical evidence not only reinforces the immunosuppressive role of copper ions in the tumor microenvironment but also provides strong support for our proposed molecular mechanism of copper/AMPK/c‐MYC pathway‐mediated tumor immune evasion (Figure 6E).

## 3 Discussion

In the tumor microenvironment, macrophages are the most distributed innate immune cells [2, 50]. Due to the non‐negligible function of macrophages in antigen presentation and tumor cell phagocytosis, macrophage‐induced innate immunity in tumor therapy is believed to be as important as T cell‐mediated adaptive immunity [2, 50]. However, under the control of the tumor cells with increased CD47 expression, most tumor cells can bind to the SIRPα ligand located on the cell membrane of macrophages, which then send “don't eat me” signal to prevent macrophage phagocytosis [51, 52]. In addition, clinically widely used tumor therapy strategies, like radiotherapy and chemotherapy with cisplatin and doxorubicin, would further increase IL‐18 secretion to stimulate the activation of mTOR/c‐Myc signaling to promote CD47 expression [45, 53]. Thus, CD47 disruption may be used as an efficacious method to boost macrophage‐induced innate immunity. To better understand how the CD47 protein is regulated in tumors, we reveal a potent, undiscovered regulatory mechanism underlying CD47 upregulation in bladder tumors and breast cancers by affecting the mitochondria/AMPK/c‐MYC axis in this study (Figure 1). By realizing tumor mitochondria‐targeting copper withdrawal through using CYN‐CDA@Alb, further designed in this research, the growth of local, re‐challenged, and metastasis tumors is depressed with sensitized tumor innate and adaptive immunity (Figures 4 and 5). Our findings highlight the uncovered role of copper ions in innate immunity resistance via immune checkpoint regulation, like CD47 and PD‐L1.

Metal ions are the main components of nearly half of the biologically active enzymes, which are vital for most biological processes, especially in mitochondria [54]. In the past few years, much attention has been paid to the vital role of metal ions in innate immunity and host defense against invading bacteria, fungi, and viruses [55, 56]. Apart from this, metal ions like copper, iron, manganese, and et al. each played a unique role in sensitizing tumor immunotherapy, such as generating more reactive oxygen, inducing ferroptosis, accelerating cuproptosis, and activating the cGAS‐sting pathway [56, 57]. Thus, metal ion‐sensitized immunotherapy is believed to be a highly effective strategy for better clinical tumor therapy application [58, 59]. But, up to now, the role of most metal ions in the expression of immune checkpoints has still not been fully revealed and neglected [58, 59]. In this study, the possible role of copper depletion in affecting PD‐L1 expression via affecting mitochondria/AMPK/c‐MYC axis is revealed, which then could activate T‐cell induced congenital immunity (Figure 1). As we all know, apart from copper, most other metal ions like iron, gallium, and calcium all play vital roles in maintaining mitochondrial homeostasis, as well as inducing AMPK phosphorylation [60, 61]. When breaking the mitochondrial homeostasis by these ions, the AMPK/c‐MYC axis may be regulated to influence the PD‐L1 expression, which still needs to be further revealed. Considering the multiple functions of the AMPK pathway in affecting PD‐L1 and CD47 expression, some metal ions may affect the innate and adaptive immune‐sensitivity to boost cancer immunotherapy [60, 61]. However, we are still in the early stages of discovering the multiple immune functions of ions and understanding the mechanistic role of these ions in immune regulation.

Like tumors, innate and adaptive immune‐resistance with increased PD‐L1 and CD47 expression could also accelerate disease progression in some other high‐incidence diseases, including but not only idiopathic fibrosis, fatty liver, atherosclerosis, and aging [62, 63]. Before, it is generally assumed that metal ions like copper and iron could induce the development of these diseases via inducing reactive oxygen and an inflammatory microenvironment [63, 64]. But, just recently, it has been proved that activated fibroblasts would raise the expression levels of CD47 and PD‐L1 to limit the phagocytosis process of stimulated fibroblasts by macrophages and suppress the adaptive immunity effects of T cells [65]. Besides, the anti‐atherosclerotic effect of the drug is also enhanced when using the CD47 antibody [66]. As we proved in this study, copper ion depletion induced by CYN‐CDA@Alb nanoparticles possess the capacity to induce PD‐L1 and CD47 expression (Figure 1). Thus, copper ions may play incredibly important roles in the occurrence, progress, and deterioration of these diseases, meaning that copper ions intervention may also work as a potent strategy for some chronic progressive diseases.

## 4 Conclusion

In this study, to solve the drawbacks of traditional copper depletion agents, a tumor mitochondria‐selective copper depletion nanoparticle named CYN‐CDA@Alb is prepared. As proved, CYN‐CDA@Alb nanoparticles more effectively depress CD47 and PD‐L1 expression by inducing AMPK phosphorylation, which then reverses innate and adaptive immune resistance to increase T cell killing capacity and macrophage phagocytosis ability to tumor cells. Following this, loss of “don't find me” PD‐L1 and “don't eat me” CD47 signals induced by CYN‐CDA@Alb nanoparticles depress local tumor growth, inhibit tumor metastasis, and slow abscopal tumor growth. Meanwhile, CYN‐CDA@Alb‐mediated copper depletion also sensitizes some other clinically widely used tumor therapy strategies like radiotherapy by avoiding the possibly occurring innate and adaptive immune resistance with increased PD‐L1 and CD47. All in all, our study reveals the possibility of using copper depletion nanoparticle CYN‐CDA@Alb to awaken the innate and adaptive immune system, which may open up a new avenue for applying the copper disruption strategy as an adjunctive immunotherapy for cancer patients.

## 5 Experimental Section

### 5.1 Materials

The antibodies and reagents used in this research were as follows: β‐Actin (#AF7018, Affinity), PD‐L1 (#DF6526, Affinity; #ab205921, Abcam), CD47 (#DF6649, Affinity; #ab218810, Abcam), p‐AMPK (#2535, Cell Signaling Technology (CST)), AMPK (#2532, CST), Ki67 (#AF0198, Affinity), IFN‐γ (#DF6045, Affinity; #ab231036, Abcam), Granzyme B/H (#AF0175, Affinity; #ab134933, Abcam), CD3 (78588S, CST; #ab16669, Abcam), CD4 (ab288724, Abcam; #ab133616, Abcam), CD8 (98941S, CST; #ab237709, Abcam), C‐MYC (#13987, CST), Bcl‐2 (#15071, CST), Cleaved‐Caspase3 (#AF7022, Affinity), N‐cadherin (#AF5239, Affinity), SLC31A1 (#ab133385, Abcam), Goat Anti‐Rabbit IgG HRP (#S0001, Affinity), CuCl_2_·2H_2_O (C805298, Macklin), Metformin (HY‐B0627, MCE), Acadesine (AICAR) (HY‐13417, MCE), BC1618 (HY‐134656, MCE), Cycloheximide (CHX) (C729197, aladdin), Ammonium tetrathiomolybdate (ATTM) (A828261, Macklin), Bis(thiobenzoyl) disulfide (DTBS) (B909295, Macklin), Tris(2‐pyridylmethyl)amine (CDA).

### 5.2 Bioinformatics Analysis

For online public data, we downloaded the expression levels of SLC31A1, CD47, and PD‐L1 in multiple cancers and the clinical data of each cancer from the TCGA database (https://www.cancer.gov/ccg/research/genome‐sequencing/tcga). The R package ggplot2, stats, car, survival, and survminer were used for analysis. IHC data for SCL31A1 were obtained from Human ALTS (https://www.proteinatlas.org/). Tumor IMmune Estimation Resource (TIMER) database was used to assist the analysis.

For private sequencing Data, after the data were standardized, we used the R language for analysis. The R packages used are: ggplot2, clusterProfiler, msigdbr, edgeR, etc.

### 5.3 Cell Culture

T24 human bladder cancer cells and 4T1 murine breast cancer cells were maintained in RPMI‐1640 medium (Gibco) supplemented with 10% fetal bovine serum (FBS) (Gibco) and 1% penicillin‐streptomycin (Gibco). MB49 murine bladder cancer cells were cultured in DMEM (Gibco) containing 10% FBS and 1% penicillin‐streptomycin. Bone marrow‐derived macrophages (BMDMs) were generated and cultured in RPMI‐1640 medium supplemented with 10% FBS, 1% penicillin‐streptomycin, and 20 ng/mL recombinant murine M‐CSF. All cells were incubated at 37°C in a humidified atmosphere with 5% CO_2_ using a standard cell incubator (Thermo Fisher Scientific, USA). MB49 cells were cultured with 100 µm CuCl_2_ for 8 h, T24 cells with 500 µm CuCl_2_ for 8 h, and 4T1 cells with 80 µm CuCl_2_ for 8 h to establish a copper‐enriched cellular microenvironment.

### 5.4 T Cell‐Mediated Tumor Cell Killing Assay

Blood samples were collected from healthy volunteers under the approved protocol of the Ethics Committee of the Second Xiangya Hospital, following the Helsinki Declaration. Peripheral blood mononuclear cells (PBMCs) were isolated using a Lymphocyte Separation Tube. T cells were purified from PBMCs with EasyStep Human T Cell Isolation Cocktail, Dextran RapidSpheres (40 µL/mL, Stemcell), and an isolation magnet (Stemcell). T cells were cultured in ImmunoCult‐XF T Cell Expansion Medium and activated with ImmunoCult Human CD3/CD28/CD2 T Cell Activator (Stemcell). T cells were co‐cultured with drug‐pretreated T24 cells (12:1 ratio), treated with anti‐CD3 antibody and IL‐2. After 24 h, tumor cells were stained with crystal violet, and T cells were washed with PBS.

### 5.5 Evaluation of Macrophage Phagocytosis of Cancer Cells In Vitro

MB49 and 4T1 tumor cells were labeled with dye CellTrace Oregon Green 488 (#C34555, ThermoFisher) and incubated with different concentrations of CuCl_2_ for 8 h. The tumor cells were further co‐cultured with F4‐80‐labeled (#14‐4801‐85, ThermoFisher) BMDM for 1 h, and the phagocytosis of macrophages was detected by flow cytometry.

### 5.6 Real‐Time Quantitative PCR (RT‒qPCR)

Total cellular RNA was extracted using TRIzol reagent (Invitrogen), and cDNA was synthesized by Takara's reverse transcription kit (#RR047A). Subsequently, the mRNA expression levels of the target genes were measured using a real‐time quantitative PCR kit (#RR820A, Takara, Japan). The primer sequences used are listed in Table S1.

### 5.7 Protein Half‐Life Assays

Cells were treated in pre‐warmed medium containing a final concentration of 50 µg/mL CHX, samples were collected at preset times, total protein was extracted, and target protein levels were analyzed by western blot assay. The control group was treated with PBS, and the experimental group was treated with 100 micromoles of CuCl_2_.

### 5.8 Cellular Thermal Shift Assay

To evaluate protein thermal stability, cell lysates were incubated at different temperatures (37–70°C) for 3 min, followed by cooling on ice and centrifugation. The supernatants were analyzed by western blot to assess the levels of soluble target protein at each temperature, and thermal denaturation curves were generated accordingly. The control group was treated with PBS, and the experimental group was treated with 100 micromoles of CuCl_2_.

### 5.9 Drug Affinity Responsive Target Stability

DARTS was conducted to determine the direct interaction between CuCl_2_ and the target protein. Cell lysates were incubated with CuCl_2_ and a vehicle control, followed by limited proteolysis using pronase. After digestion, samples were resolved by SDS‐PAGE and analyzed by western blot. The control group was treated with PBS, and the experimental group was treated with 100 micromoles of CuCl_2_.

### 5.10 Transfection

Cells were infected with lentivirus encoding specific shAMPKα1, shAMPKα2, and then incubated for 24 h in the presence of 5 µg/mL Polybrene before being replaced with fresh medium. Stable transfected cell lines were screened by adding 2 µg/mL puromycin at 48 h after infection. After continuous screening for 5–7 days, the interference efficiency of target genes was detected by western blot.

### 5.11 Synthesis of CYN and CYN‐CDA

CYN (CAS Number: 199444‐11‐6) and N1,N1‐Bis(pyridin‐2‐ylmethyl)ethane‐1,2‐diamine (CAS Number: 189440‐33‐3) were obtained from Macklin Inc. Generally, CYN (519 mg), N1,N1‐Bis(pyridin‐2‐ylmethyl)ethane‐1,2‐diamine (726 mg), and anhydrous K_2_CO_3_ (138 mg) were dissolved in anhydrous acetonitrile. The solution was then heated to 85°C and stirred for 6 h. The final product of CYN‐CDA was purified by silica column chromatography. Characterization data for CYN‐CDA: HR‐MS (ESI‐TOF) calcd for C_46_H_53_ClN_6_ [M ‐Cl]^+^ 689.4327, found 689.4316.

### 5.12 Preparation of CYN@Alb and CYN‐CDA@Alb

5 mg of CYN and CYN‐CDA were dissolved in 0.5 mL of dimethyl sulfoxide (DMSO). 300 mg of bovine serum albumin (BSA, Sigma–Aldrich) was dissolved in 60 mL of ultrapure water, pre‐prepared solutions of CYN and CYN‐CDA were added, and the mixture was stirred for 2 h for self‐assembly. Then, DMSO was removed by using a 50 mL Amicon ultrafilter (30 kDa membrane), and the dye‐protein complexes (CYN and CYN‐CDA) were concentrated to 10 mL. To form stable nanoparticles, the concentrated solution was heated at 70°C for 10 min, followed by cooling to room temperature.

### 5.13 Characterization of Nanoparticles

The optical properties of the compounds and their nanoparticles were characterized by UV‐visive‐near infrared spectrophotometer (CARY 5000, Agilent, USA) and a near‐infrared fluorescence spectrometer (Horiba Fluoromax‐4). For NIR fluorescence determination, the excitation wavelength was set at 580 nm, the emission spectral range was 600–800 nm, the slit width was 5 nm, and the scan rate was 600 nm/min. The mean particle size and Zeta potential of the nanoparticles were analyzed by dynamic light scattering (DLS, Zetasizer Nano ZS ZEN3600) and field emission scanning electron microscopy (SU8010, HITACHI).

To evaluate the stability of the nanoparticles, CYN‐CDA@Alb nanoparticles were dispersed in PBS or serum, stored at room temperature, and size changes were detected using DLS at different time points. In addition, CYN‐CDA@Alb lyophilized powder was stored at ‐80°C for 3 months and subsequently re‐dissolved in pure water, and its particle size was measured by DLS.

### 5.14 Protein Binding Assay

To explore the effect of Alb on the fluorescence characteristics of CYN‐CDA, CYN‐CDA was dissolved in PBS buffer containing 0%–10% Alb at a final concentration of 2 µm, and detected by a near‐infrared fluorescence spectrometer. To further evaluate its binding sites on the Alb molecule, the molecules with specific binding sites of Alb (warfarin, ibuprofen, digoxin, and quinidine) were added to 5% Alb (189 µm) and incubated at room temperature for 30 min, followed by the addition of 2 µm CYN‐CDA and continued incubation for another 30 min. Subsequently, a fluorescence signal assay was performed to determine the distribution of CYN‐CDA binding sites.

### 5.15 Molecular Calculation of Protein Binding Capacity

The molecular structure of CYN‐CDA was imported into ChemBio3D Ultra 14.0 for energy minimization, with the minimum RMS gradient set to 0.001. The optimized structure was saved in mol2 format. The molecule was then processed in AutoDockTools 1.5.6 for hydrogen addition, charge calculation (Gasteiger charges), and rotatable bond assignment, and subsequently saved in pdbqt format. The crystal structure of human serum albumin (ALB, PDB ID: 1N5U) was downloaded from the Protein Data Bank (PDB). All crystallographic water molecules and original ligands were removed using PyMOL 2.3.0. The cleaned protein structure was imported into AutoDockTools (v1.5.6) for hydrogen addition, charge assignment, and atom type specification, and then saved in pdbqt format. The potential binding pockets of ALB were predicted using POCASA 1.1. Molecular docking was performed with AutoDock Vina 1.1.2. The docking grid box was centered at x = 29.0, y = 10.8, z = 15.0, with dimensions of 60 × 60 × 60 (grid spacing: 0.375 Å), and all other parameters were set to default. The binding interactions were analyzed and visualized using PyMOL 2.3.0 and LigPlot+ v2.2.8.

### 5.16 Subcellular Localization of CYN‐CDA@Alb

MB49 cells were incubated with either CYN@Alb or CYN‐CDA@Alb (both at 2 µm) for 4 h. After incubation, cells were washed with PBS and subsequently stained with MitoTracker Green (100 nm) for 30 min. Excess dye was removed by PBS washing. Intracellular fluorescence was then visualized using a confocal laser scanning microscope (CLSM, IVIS Lumina XRMS Series III).

### 5.17 Detection of Mitochondrial Membrane Potential by JC‐1 Probe

MB49 cells (2 × 10^5^) were seeded into confocal culture dishes and incubated overnight. The next day, cells were treated with CYN@Alb (2 µm), CDA (2 µm), or CYN‐CDA@Alb (2 µm) (calculated by CYN, CDA, or CYN‐CDA concentration) for 4 h, with untreated cells in complete medium serving as the negative control. After 24 h of culture, JC‐1 dye was added to the medium, and cells were incubated for an additional 30 min. Mitochondrial membrane potential was then assessed using CLSM.

### 5.18 ROS Detection

The ROS levels in the cells treated with different drugs were evaluated using the ROS probe DCFH‐DA (2’,7’‐dichlorodihydrofluorescein diacetate). Cells were treated with drugs for 12 h, probes were added and incubated for 20 min, followed by imaging analysis by CLSM.

### 5.19 Live/Dead Cell Staining Assay

Cells were seeded on confocal plates and incubated overnight, and subsequently treated with CDA (2 µm), CYN@Alb (2 µm), or CYN‐CDA@Alb (2 µm). After treatment for 24 h, the cells were washed with PBS and then stained with Calcein‐AM and PI for 30 min. Finally, the fluorescence signal was observed by CLSM to determine the cell survival and death.

### 5.20 Measurement of ADP/ATP Ratio

After an overnight culture of 1 × 10^4^ MB49 cells in 24‐well plates, cells were exposed to different drug treatments for 12 h. Subsequently, cell lysates were prepared, and ADP/ATP levels were measured using the ADP/ATP‐lite kit (T008, Vigorous Biotec).

### 5.21 Cytotoxicity Assay

Various tumor cell lines (MB49, T24, and 4T1) were subjected to cytotoxicity evaluation. Cells (5 × 10^3^/well) were plated in 96‐well plates and incubated for 24 h, followed by exposure to serial concentrations of CDA, CYN@Alb, or CYN‐CDA@Alb for 12 h. After replacing with fresh medium, cell viability was measured using the CCK‐8 assay, and IC50 values were determined accordingly.

### 5.22 Immunofluorescence (IF) Assay

MB49 cells (2 × 10^5^) were seeded in confocal culture dishes and incubated overnight. The next day, cells were treated with CDA (2 µm), CYN@Alb (2 µm), or CYN‐CDA@Alb (2 µm) for 24 h, followed by two washes with PBS. Cells were then fixed with 4% paraformaldehyde at room temperature for 15 min and permeabilized with 0.1% Triton X‐100 for 5 min. To block nonspecific binding sites, cells were incubated in 5% bovine serum albumin in PBS for 1 h. Subsequently, cells were incubated overnight at 4°C with primary antibodies against PD‐L1 or CD47 (1:200, Affinity) diluted in blocking buffer. After three washes with PBS, cells were incubated with Alexa Fluor 488‐conjugated goat anti‐rabbit secondary antibody (A‐11008, Invitrogen) in the dark at room temperature for 1 h. Following another three PBS washes, nuclei were stained with DAPI for 5 min, and cells were rinsed twice with PBS. Confocal laser scanning microscopy (CLSM) was used to visualize the expression and subcellular localization of PD‐L1 and CD47.

### 5.23 The Wound Healing (Cell Scratch) Assay

Cells were seeded into 6‐well plates and cultured until reaching 90%–100% confluence. A sterile 200 µL pipette tip was used to make a straight scratch (wound) through the cell monolayer. Detached cells were removed by gently washing with PBS. Then, cells were incubated in serum‐free medium containing different treatments as indicated. Images of the wound area were captured at 0 and 24 h using an inverted microscope. The migration distance was quantified by measuring the gap width at each time point, and wound closure was calculated to assess cell migratory ability.

### 5.24 Transwell Invasion Assay

The invasive capacity of tumor cells was evaluated using Transwell chambers (8 µm pore size; Corning) pre‐coated with Matrigel (BD Biosciences). Briefly, matrigel was diluted with serum‐free medium and applied to the upper surface of the insert membrane, followed by incubation at 37°C for 1–2 h to allow gelation. Tumor cells (e.g., 1 × 10^5^ cells/well) suspended in serum‐free medium containing the indicated treatments were seeded into the upper chamber. The lower chamber was filled with medium containing 10% FBS as a chemoattractant. After incubation at 37°C for 24 h, non‐invading cells on the upper membrane surface were gently removed using a cotton swab. Cells that had invaded through the matrigel and migrated to the underside of the membrane were fixed with 4% paraformaldehyde for 15 min and stained with crystal violet for 10–20 min. The number of invaded cells was counted under a microscope in at least five randomly selected fields.

### 5.25 Clone Formation Assay

To evaluate the effect of drugs on tumor cell proliferation, a colony formation assay was performed. Tumor cells were seeded into 6‐well plates at a density of 500–1000 cells per well and allowed to adhere overnight. Cells were then treated with the indicated drugs for 24 h. After drug exposure, the medium was replaced with fresh complete medium, and cells were cultured for 10–14 days to allow colony formation. The medium was changed every 3–4 days. At the end of the incubation period, colonies were fixed with 4% paraformaldehyde for 15 min and stained with 0.1% crystal violet for 15–30 min. Colonies containing more than 50 cells were counted under a microscope. The colony numbers were used to evaluate the long‐term proliferative capacity of tumor cells.

### 5.26 Animal Ethics and Feeding

All animal studies were approved by the Institutional Animal Care and Use Committee (IACUC) of Wenzhou Institute, University of Chinese Academy of Sciences (Protocol No. WIUCAS24080703 and WIUCAS24091906). Female C57BL/6 and BALB/c mice (4–6 weeks old, 18–20 g) were obtained from the Zhejiang Experimental Animal Center and housed in the SPF facility of the Wenzhou Institute under standard conditions (24°C, 12 h light/dark cycle). Mice had free access to autoclaved water and sterile chow. All procedures involving animals were carried out in accordance with the guidelines for the care and use of laboratory animals published by the U.S. National Institutes of Health (Bethesda, MD, USA).

For xenograft studies, tumor growth was monitored using digital calipers, and tumor volume was calculated using the formula: (length × width^2^)/2. Injections were performed under sterile conditions within biosafety cabinets. At the endpoint, mice were humanely euthanized, and tumor xenografts were harvested for subsequent analyses.

### 5.27 In Vitro Hemolysis Assay of CYN‐CDA@Alb

The hemolytic activity of CYN‐CDA@Alb was assessed using a standard in vitro red blood cell (RBC) lysis assay. Briefly, 40 µL of freshly collected RBC suspension was incubated with 100 µL of CYN‐CDA@Alb at varying concentrations (0, 0.1, 0.5, 1.0, and 5 µm, based on CYN‐CDA). Distilled water served as the positive control. The mixtures were incubated at 37°C for 2 h without disturbance. After incubation, samples were centrifuged to pellet intact RBCs, and the supernatants were collected. The absorbance of the supernatants at 540 nm, corresponding to released hemoglobin from lysed RBCs, was measured using a microplate reader to determine the extent of hemolysis.

### 5.28 In Vivo Biocompatibility Assessment

C57BL/6 mice were randomly assigned to receive intravenous injections of CYN‐CDA@Alb at doses of 2 or 5 mg/kg, or an equal volume of blank vehicle as a control. Blood samples were collected from the retro‐orbital sinus at 24 and 48 h post‐injection. Serum was obtained by centrifugation and analyzed for biochemical markers, including serum creatinine (CR), blood urea nitrogen (BUN), alanine aminotransferase (ALT), and aspartate aminotransferase (AST), using commercial assay kits (Nanjing Jiancheng Bioengineering Institute, China) based on enzymatic methods.

At the study endpoint, mice treated with either 5 mg/kg CYN‐CDA@Alb or PBS were euthanized. Major organs (heart, liver, spleen, lung, and kidney) were carefully harvested, fixed in 10% formalin, embedded in paraffin, sectioned, and stained with hematoxylin and eosin (H&E) for histopathological evaluation of potential tissue damage.

### 5.29 In Vivo Fluorescence Imaging and Bio‐Distribution Study

To establish the MB49 tumor xenograft model, 5 × 10^5^ MB49 tumor cells suspended in 100 µL PBS were subcutaneously injected into the right flank of C57BL/6 mice. Once the tumor volume reached approximately 150 mm^3^, mice were administered CYN‐CDA@Alb via intravenous injection at a dose equivalent to 2 mg/kg of CYN‐CDA.

At designated time points (0, 12, and 48 h post‐injection), in vivo fluorescence imaging was performed using the IVIS Lumina system. Prior to imaging, mice were anesthetized via intraperitoneal injection of 2% pentobarbital sodium. After the final imaging at 48 h, mice were sacrificed, and major organs (heart, liver, spleen, lungs, kidneys, intestines, and muscle) along with tumor tissues were excised for ex vivo fluorescence imaging to assess bio‐distribution.

### 5.30 Protein Extraction from Tumor Tissues

Tumor tissues were harvested from euthanized mice and immediately snap‐frozen in liquid nitrogen. The frozen samples were pulverized into fine powder using a pre‐chilled mortar and pestle under liquid nitrogen to prevent protein degradation. The powdered tissue was then lysed in ice‐cold RIPA lysis buffer (containing protease and phosphatase inhibitors) and incubated on ice for 30 min with intermittent vortexing. Following lysis, the homogenates were centrifuged at 12 000 rpm for 15 min at 4°C to remove debris. The supernatants containing total protein were collected, and the protein concentrations were determined using a BCA Protein Assay Kit (Thermo Fisher Scientific).

### 5.31 H&E Staining and Immunohistochemistry Assay

At the end of the experiment, tumor tissues were harvested from euthanized mice, fixed in 10% neutral buffered formalin for at least 24 h, and then embedded in paraffin. Tissue blocks were sectioned at a thickness of 4 µm using a microtome and mounted on glass slides.

For hematoxylin and eosin (H&E) staining, tissue sections were deparaffinized in xylene, rehydrated through a graded ethanol series, stained with hematoxylin for 5 min, rinsed in running water, differentiated with acid alcohol, and counterstained with eosin. After dehydration and mounting, the sections were observed under a light microscope to assess histological features.

For immunohistochemistry, sections were first deparaffinized, rehydrated, and subjected to heat‐induced antigen retrieval in citrate buffer (pH 6.0) using a microwave or water bath. Endogenous peroxidase activity was blocked by incubation with 3% hydrogen peroxide for 10 min. Non‐specific binding was blocked using 5% bovine serum albumin (BSA) in PBS for 30 min at room temperature.

The sections were then incubated overnight at 4°C with primary antibodies (e.g., anti‐PD‐L1, anti‐CD47, anti‐CD8, etc.) at appropriate dilutions. After washing, sections were incubated with HRP‐conjugated secondary antibodies for 30 min at room temperature. Color development was achieved using DAB substrate, followed by hematoxylin counterstaining. Slides were dehydrated, mounted, and visualized under a bright‐field microscope.

### 5.32 In Vivo Flow Cytometry Assay

Following enzymatic dissociation of the tumor tissue, red blood cells were eliminated using ACK lysis buffer. The resulting single‐cell suspension was then incubated with FITC‐conjugated anti‐mouse CD3 (Invitrogen, #2527364), BV421‐conjugated anti‐mouse CD4 (Invitrogen, #2599066), and APC‐conjugated anti‐mouse CD8 (BioLegend, #100712) antibodies. Labeled cells were subsequently subjected to flow cytometric analysis.

### 5.33 Proteomics Profiling and Bioinformatic Analysis

Tumor tissues from both control and treatment groups were harvested and snap‐frozen in liquid nitrogen. Total RNA was extracted using TRIzol reagent (Invitrogen) following the manufacturer's instructions. RNA integrity and concentration were assessed using a NanoDrop spectrophotometer and an Agilent 2100 Bioanalyzer. RNA sequencing and subsequent bioinformatics analyses were performed by Shanghai OE Biotech Co., Ltd.

### 5.34 Mouse Anti‐Tumor Copper Chelation Therapy Model

To evaluate the therapeutic efficacy of CDA in vivo, a subcutaneous tumor model was established in C57BL/6 mice. Briefly, 5 × 10^5^ MB49 cells suspended in 100 µL PBS were inoculated into the right flank of each mouse. Tumor volume was monitored every other day using a digital caliper and calculated as (length × width^2^)/2. Once the tumors reached approximately 50 mm^3^ (on day 7), mice were randomly divided into three groups: (1) intravenous injection of PBS, (2) intravenous injection of CDA (2 mg/kg), and (3) intratumoral injection of CDA (1 mg/kg). Treatments were administered on day 0, day 3, and day 7. During the treatment period, mouse body weight and tumor volume were recorded every two days to assess the treatment response and potential toxicity.

### 5.35 Mouse Anti‐Tumor Immunotherapy Model

To evaluate the anti‐tumor efficacy of various treatments, a subcutaneous tumor model was established using C57BL/6 mice. Briefly, 5 × 10^5^ MB49 cells suspended in 100 µL PBS were inoculated subcutaneously into the right flank of each mouse. Once the tumor volume reached approximately 50 mm^3^ (defined as day ‐8), mice were randomly divided into the following treatment groups: (1) PBS, (2) CDA (2 mg/kg), (3) CYN@Alb (2 mg/kg), (4) anti‐CD47 (3 mg/kg, #BE0270, BioXCell), (5) anti‐PD‐L1 (3 mg/kg, #BE0101, BioXCell), (6) anti‐CD47 + anti‐PD‐L1 (3 mg/kg each), and (7) CYN‐CDA@Alb (2 mg/kg). Treatments were administered via tail vein injection on days 0, 3, 6, and 9. Tumor size was measured every other day using a digital caliper, and tumor volume was calculated as (length × width^2^)/2. Body weight was also recorded every two days to monitor systemic toxicity.

### 5.36 Mouse Anti‐Tumor Immune Memory

To investigate the systemic anti‐tumor effects of various formulations, a bilateral 4T1 breast cancer model was established in female BALB/c mice. Briefly, 8 × 10^5^ 4T1 cells suspended in 100 µL PBS were subcutaneously inoculated into the right upper axilla of BALB/c mice. When the tumor volume reached approximately 50 mm^3^ (defined as day ‐7), mice were randomly divided into the following groups: (1) PBS, (2) CDA (2 mg/kg), (3) CYN@Alb (2 mg/kg), and (4) CYN‐CDA@Alb (2 mg/kg). Drugs were administered via tail vein injection on days 0, 3, 6, and 9. On day 14, the primary tumors in the right axilla were surgically removed under anesthesia. After a recovery period, a second tumor was established by injecting 8 × 10^5^ 4T1 cells into the left lower groin region on day 18. Tumor volume and body weight were recorded every other day throughout the study. Mice were euthanized on day 32, and tumors were harvested for further analysis. Tumor volume was calculated using the formula: (length × width^2^)/2.

### 5.37 Mouse Anti‐Tumor RT Enhancement Model

To evaluate the combined effect of CYN‐CDA@Alb and radiotherapy, a bilateral MB49 bladder tumor model was established in C57BL/6 mice. On day ‐8, 5 × 10^5^ MB49 cells suspended in 100 µL PBS were subcutaneously injected into the right upper axilla to generate a primary (irradiated) tumor. On day ‐1, an equal number of MB49 cells were inoculated into the left lower groin to form a distal (non‐irradiated) tumor.

Mice were randomly divided into four groups: Group A: PBS treatment without radiotherapy. Group B: CYN‐CDA@Alb (0.5 mg/kg) treatment without radiotherapy. Group C: PBS treatment with radiotherapy. Group D: CYN‐CDA@Alb (0.5 mg/kg) treatment with radiotherapy.

Drug treatments were administered via tail vein injection on days 0, 3, 6, and 9. For groups receiving radiotherapy (Groups C and D), the primary tumors were locally irradiated (3 Gy per session, 6 Gy/min) on days 1, 4, 7, and 10 using a small animal irradiator.

On day 14, the irradiated (primary) tumors were surgically removed under anesthesia. On day 24, the distal tumors were excised and harvested for further analysis. Tumor volume and mouse body weight were recorded every other day throughout the experiment. Tumor volume was calculated using the formula: volume = (length × width^2^)/2.

### 5.38 Mouse Anti‐Tumor Survival Analysis

To evaluate the anti‐tumor efficacy of various formulations, a subcutaneous tumor model was established in C57BL/6 mice. A total of 5 × 10^5^ MB49 bladder cancer cells suspended in 100 µL PBS were injected subcutaneously into the right flank of each mouse. When the tumors reached approximately 50 mm^3^ in volume (defined as day ‐7), mice were randomly divided into four groups and administered intravenous injections of either PBS, CDA (2 mg/kg), CYN@Alb (2 mg/kg), or CYN‐CDA@Alb (2 mg/kg) on days 0, 3, 6, and 9. Tumor volume and body weight were measured every other day using a digital caliper to monitor tumor progression and systemic toxicity. Tumor volume was calculated as (length × width^2^)/2. The experiment was terminated when the tumor volume reached 2000 mm^3^ or when animals met humane endpoint criteria.

To assess the therapeutic efficacy of CYN‐CDA@Alb in combination with radiotherapy, a subcutaneous MB49 tumor model was established in C57BL/6 mice. Briefly, 5 × 10^5^ MB49 bladder cancer cells suspended in 100 µL PBS were injected subcutaneously into the right flank of each mouse. When tumor volumes reached approximately 50 mm^3^ (defined as day ‐7), mice were randomly assigned to receive intravenous injections of PBS, CYN‐CDA@Alb (2 mg/kg), PBS combined with radiotherapy, or CYN‐CDA@Alb (2 mg/kg) combined with radiotherapy. Treatments were administered via tail vein injection on days 0, 3, 6, and 9. For the radiotherapy groups, local tumor irradiation was delivered using a 3 Gy dose (radiation efficiency: 6 Gy/min) following each injection. Tumor dimensions and body weights were recorded every other day. Tumor volume was calculated as (length × width^2^)/2. The experiment was terminated when tumor volumes reached 2000 mm^3^ or mice met humane endpoint criteria.

### 5.39 Mouse Anti‐Tumor Lung Metastasis Model

To evaluate the anti‐tumor and anti‐metastatic efficacy of CYN‐CDA@Alb, an orthotopic 4T1 breast cancer model was established using BALB/c mice. Briefly, 5 × 10^5^ 4T1 cells suspended in 100 µL PBS were inoculated subcutaneously into the right axilla of each mouse. When tumor volumes reached approximately 50 mm^3^ (defined as day ‐7), mice were randomly assigned to receive intravenous administration of PBS, CDA (2 mg/kg), CYN@Alb (2 mg/kg), or CYN‐CDA@Alb (2 mg/kg) on days 0, 3, 6, and 9. Tumor size and body weight were monitored every other day. On day 14, primary tumors were surgically removed under anesthesia. After postoperative recovery, mice were maintained until day 33, at which point lung tissues were harvested for further analysis of metastatic burden.

### 5.40 Human Tissue Specimen Collection and Immunohistochemistry Staining

Human bladder cancer tissue samples were collected from 22 patients who underwent surgery at The Second Xiangya Hospital, with prior approval from the Institutional Ethics Committee (Approval No.: 202201007). All participants provided informed consent before sample collection. Formalin‐fixed, paraffin‐embedded tumor tissues were sectioned and subjected to standard immunohistochemical staining procedures.

Quantification of IHC staining was performed based on a semi‐quantitative scoring system combining staining intensity and the percentage of positive cells. The staining intensity was scored on a scale of 0 to 3 (0 = negative, 1 = weak, 2 = moderate, 3 = strong), and the proportion of positively stained cells was scored from 0 to 5 (0 = 0%, 1 = <1%, 2 = 1%–10%, 3 = 11%–33%, 4 = 34%–66%, 5 = 67%–100%). The final IHC score was calculated as the sum of these two components, resulting in a total score ranging from 0 to 8.

### 5.41 Statistical Analysis

Statistical analysis and graphing were performed using GraphPad Prism software (version 9.0, San Diego, CA, USA). Data were presented as mean ± standard deviation (SD). Comparisons between two groups were carried out using a two‐tailed Student's *t‐*test, while multiple group comparisons were analyzed by one‐way ANOVA. A *p*‐value less than 0.05 was considered statistically significant (^*^ *p* < 0.05); ^**^ *p* < 0.01 and ^***^ *p* < 0.001 indicated stronger levels of significance; ns denotes no statistically significant difference (*p* > 0.05).

## Author Contributions

Zaigang Zhou, Rui Cheng, Ke Li, Yuan Li, and Jianliang Shen conceived and designed the research. Zaigang Zhou, Rui Cheng, Ke Li, Feiyu Liu, Xuelan Li, Zhengxiang Wang, Sheng Wu, and Huan Ding executed the experiments and wrote the manuscript. Xuelan Li, Lei Yi, Zhengxiang Wang, and Huan Ding analyzed and organized the data. Yuan Li and Jianliang Shen revised the manuscript. Yuan Li and Jianliang Shen supervised the research progress and provided professional advice.

## Conflicts of Interest

The authors declare no conflicts of interest.

## Supporting information




**Supporting File**: advs75002‐sup‐0001‐SuppMat.docx.

## Data Availability

The paper and supplemental materials provide all relevant data that support the study's findings.
