# Supplementary material for: Copper Depletion Nanoparticles Potentiate Cancer Immunotherapy by Avoiding Innate and Adaptive Immune Resistance
Source: Adv Sci (Weinh). 2026 Apr 2;13(32):e24150. doi: 10.1002/advs.202524150 (PMC13252637; doi:10.1002/advs.202524150)
Supplement: Supplementary file 1 — Supporting File: advs75002‐sup‐0001‐SuppMat.docx. [file ADVS-13-e24150-s001.docx]

**Supporting Information**

**Copper Depletion Nanoparticles Potentiate Cancer Immunotherapy by Avoiding Innate and Adaptive Immune Resistance**

Zaigang Zhou ^1, #^, Ke Li ^1,2, #^, Xuelan Li ^5^, Lei Yi ^2^, Zhengxiang Wang ^1^, Huan Ding ^1^, Sheng Wu ^1^, Feiyu Liu ^1^, Yuan Li ^2,*^, Rui Cheng ^3,*^, Jianliang Shen ^1, 4,^^*^

Z. Zhou, Z. Wang, H. Ding, S. Wu, F. Liu, J. Shen

Zhejiang Key Laboratory of Ophthalmic Drug Discovery and Medical Device Research, Eye Hospital, Wenzhou Medical University, Wenzhou, Zhejiang 325027, China.

E-mail: [shenjl@wiucas.ac.cn](mailto:shenjl@wiucas.ac.cn), sjl1@wmu.edu.cn (J. Shen)

K. Li, L. Yi, Y. Li

Department of Urology, The Second Xiangya Hospital, Central South University, Changsha, Hunan 410011, China.

E-mail: yuanlixy@csu.edu.cn (Y. Li)

R. Cheng

Department of hepatobiliary surgery, Fujian Medical University Union Hospital, Fuzhou, Fujian, China.

E-mail: [ruichengunion@126.com](mailto:ruichengunion@126.com) (R. Chen)

J. Shen

Zhejiang Engineering Research Center for Tissue Repair Materials, Wenzhou Institute, University of Chinese Academy of Sciences, Wenzhou, Zhejiang 325001, China.

X. Li

Department of Clinical Nursing, Xiangya Hospital, Central South University, Changsha, Hunan 410008, China.

^#^ These authors contributed equally to this paper as first authors. * Corresponding authors

**Supplementary Tables and Figures**

| **Gene**  **(Mouse)** | **Forward primer (5′ - 3′)** | **Reverse primer (5′ - 3′)** |
| --- | --- | --- |
| β-actin | GGCTGTATTCCCCTCCATCG | CCAGTTGGTAACAATGCCATGT |
| NBS1 | GCAGAAGACGACGAAGAGGAACAG | GCCAATCCAATCTCCGCTTCAGG |
| MRE11 | TCGGGCACAACATCTAGCAAACG | GAAGCAAAACCGGACTAATGTCT |
| RAD50 | CGATGCAGTGCTGGACAGAAGG | AGACTGACCTTTTCACCATGC |
| CD47 | CAGCGATTGGATTAACCTCCT | TGGTATACACGCCGCAATAC |
| PD-L1 | ACCAGCACACTGAGAATCAAC | GGTAGTTCTGGGATGACCAATTC |

**Table S1. The primer sequences for RT-qPCR.**


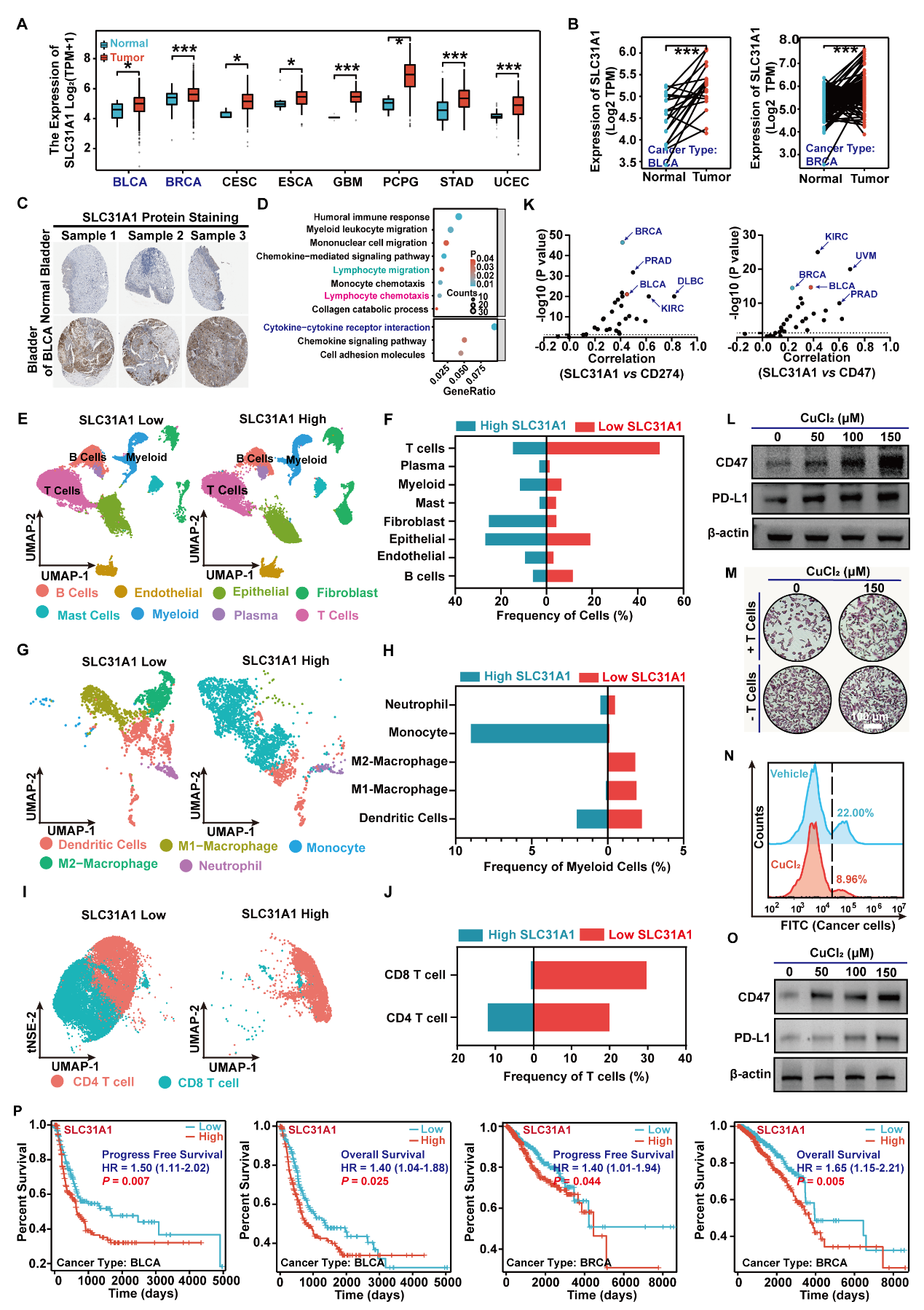
**Figure S1.** Oncogenic and immune-regulatory roles of SLC31A1 under copper ion exposure across cancers. (A) Differential expression analysis of SLC31A1 across various cancer types and normal tissues in pan-cancer datasets. (B) Paired sample analysis of SLC31A1 expression in bladder cancer (BLCA) and breast cancer (BRCA) tissues compared with matched normal tissues. (C) Representative immunohistochemical staining of SLC31A1 in BLCA tissues and normal bladder tissues. (D) GO and KEGG enrichment analysis of differentially expressed genes between SLC31A1-high and SLC31A1-low groups in BLCA. (E) t-SNE plot of global cell types. (F) Bar plot showing the relative frequencies of the global cell clusters. (G) t-SNE plot of myeloid cell types. (H) Bar plot showing the relative frequencies of different myeloid cells in the global cell clusters. (I) t-SNE plot of T cells. (J) Bar plot showing the relative frequencies of different T cells in the global cell clusters. (K) Correlation analysis of SLC31A1 expression with PD-L1 and CD47 expression across pan-cancer datasets. (L) Western blot analysis of PD-L1 and CD47 expression in MB49 cells treated with increasing concentrations of CuCl_2_. (M) Assessment of CuCl_2_ treatment on T cell-mediated cytotoxicity against T24 bladder cancer cells. (N) Phagocytosis assay evaluating the impact of CuCl_2_ on bone marrow-derived macrophage (BMDM) engulfment of MB49 tumor cells. (O) Western blot analysis of PD-L1 and CD47 expression in 4T1 cells treated with increasing concentrations of CuCl_2_. (P) Kaplan-Meier survival analysis comparing overall survival (OS) and progression-free survival (PFS) between SLC31A1-high and SLC31A1-low expression groups in BLCA and BRCA cohorts. BLCA, Bladder Urothelial Carcinoma; BRCA, Breast invasive carcinoma; PRAD, Prostate Adenocarcinoma; KIRC, Kidney Renal Clear Cell Carcinoma; DLBA, Diffuse Large B-Cell Lymphoma; UVM, Uveal Melanoma. * *p* < 0.05; ** *p* < 0.01; *** *p* < 0.001.


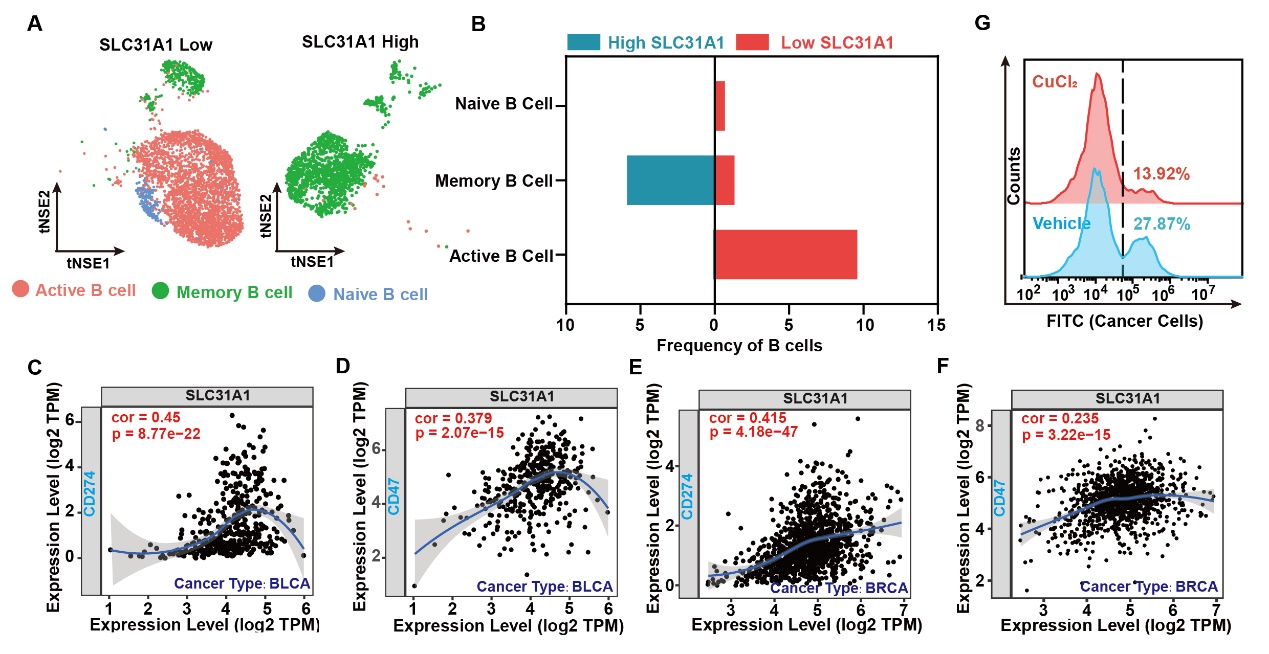


**Figure S2.** (A) t-SNE plot of B cell types. (B) Bar plot showing the relative frequencies of different B cells in the global cell clusters. (C-F) Correlation analysis of SLC31A1 expression with PD-L1 and CD47 expression across BLCA and BRCA cancers. (G) Phagocytosis assay evaluating the impact of CuCl_2_ on BMDM engulfment of 4T1 tumor cells.

**
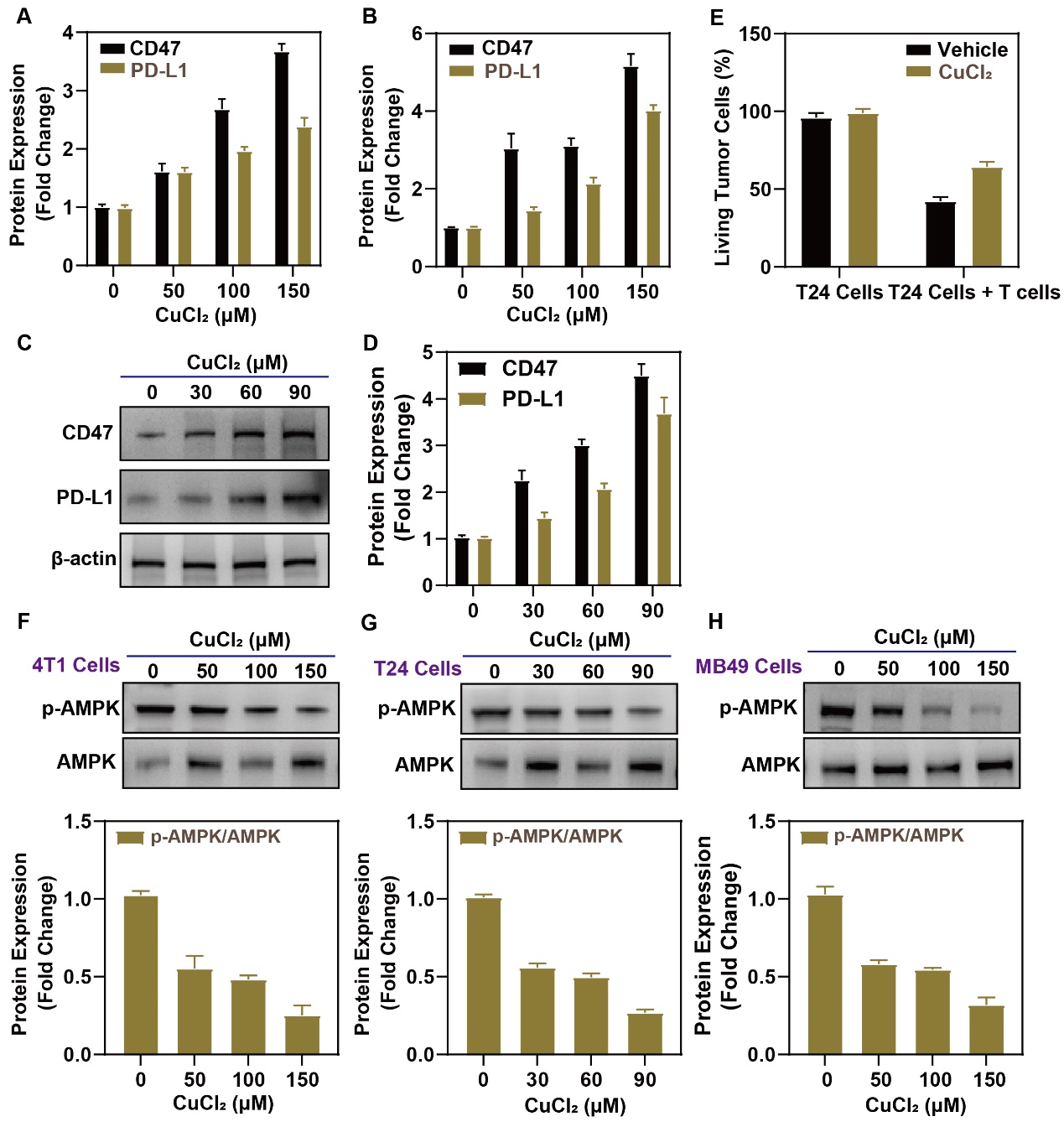
**

**Figure S3.** (A) Quantification of western blot grayscale intensity for Figure S1L (n = 3). (B) Quantification of residual tumor cell numbers following T cell-mediated cytotoxicity assay shown in Figure S1M (n = 3). (C-D) Analysis of the expression levels of PD-L1 and CD47 in T24 cells treated with different concentrations of CuCl_2_ by western blot assay, and grayscale intensity was quantified (n = 3). (E) Quantification of western blot assay grayscale intensity for Figure S1O (n = 3). (F-H) Western blot analysis of AMPK and phosphorylated AMPK (p-AMPK) expression in 4T1, T24, and MB49 cells following treatment with varying concentrations of copper ions (n = 3).

**
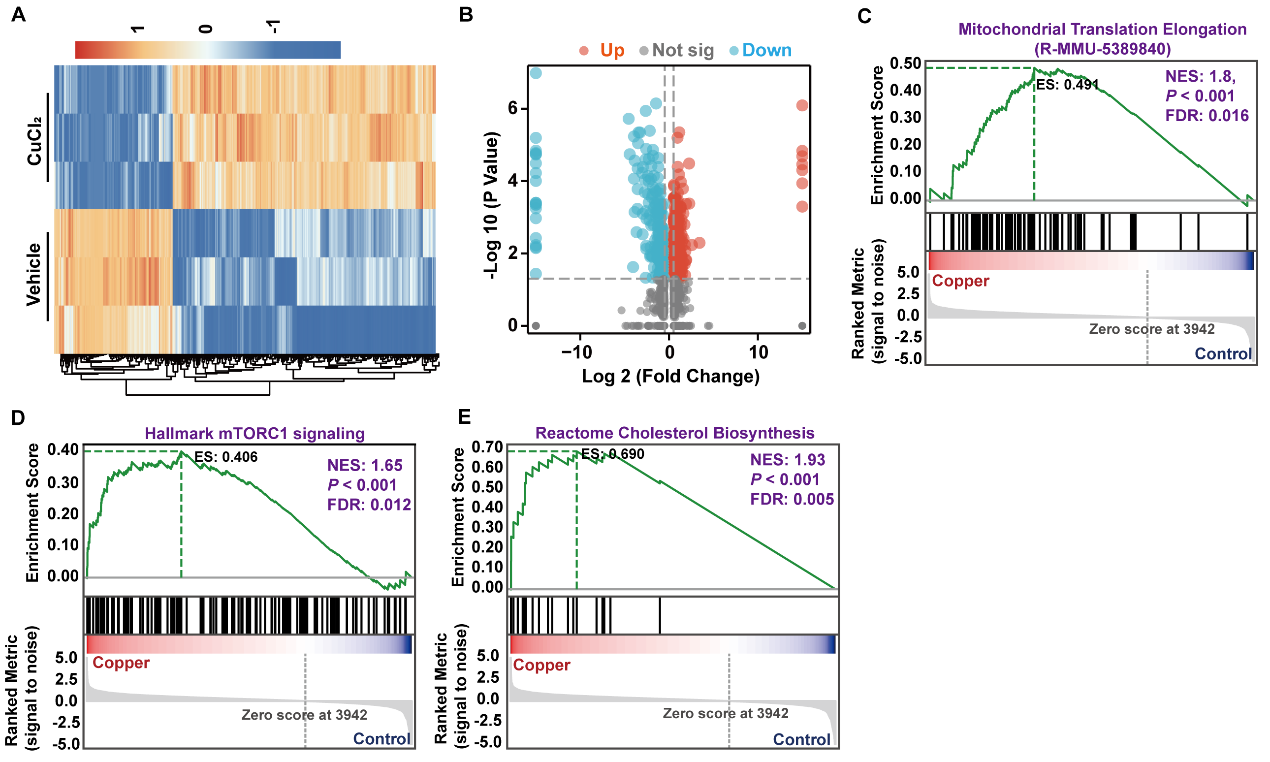
**

**Figure S4.** (A) Heatmap showing hierarchical clustering of differentially expressed proteins in MB49 cells after CuCl_2_ treatment, based on proteomic analysis. (B) Volcano plot depicting significantly upregulated and downregulated proteins following CuCl_2_ treatment in MB49 cells. (C-E) Visualization of gene set enrichment analysis (GSEA) results based on the proteomic data, highlighting key pathways altered by copper ion treatment.


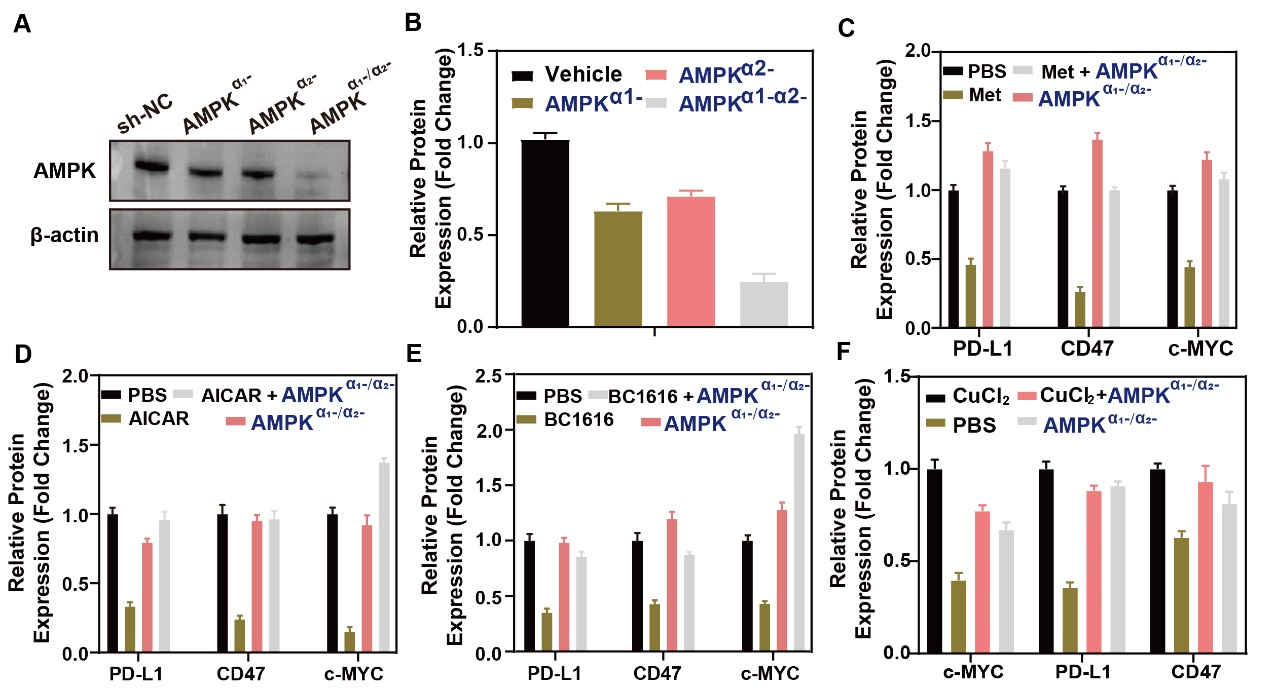


**Figure S5.** (A-B) Western blot analysis of AMPK expression in MB49 cells transfected with shRNA that targeting AMPKα1, AMPKα2, or both, and grayscale quantification (n = 3). (C-F) Quantification of western blot grayscale intensity for Figure 1H-1I (n = 3).


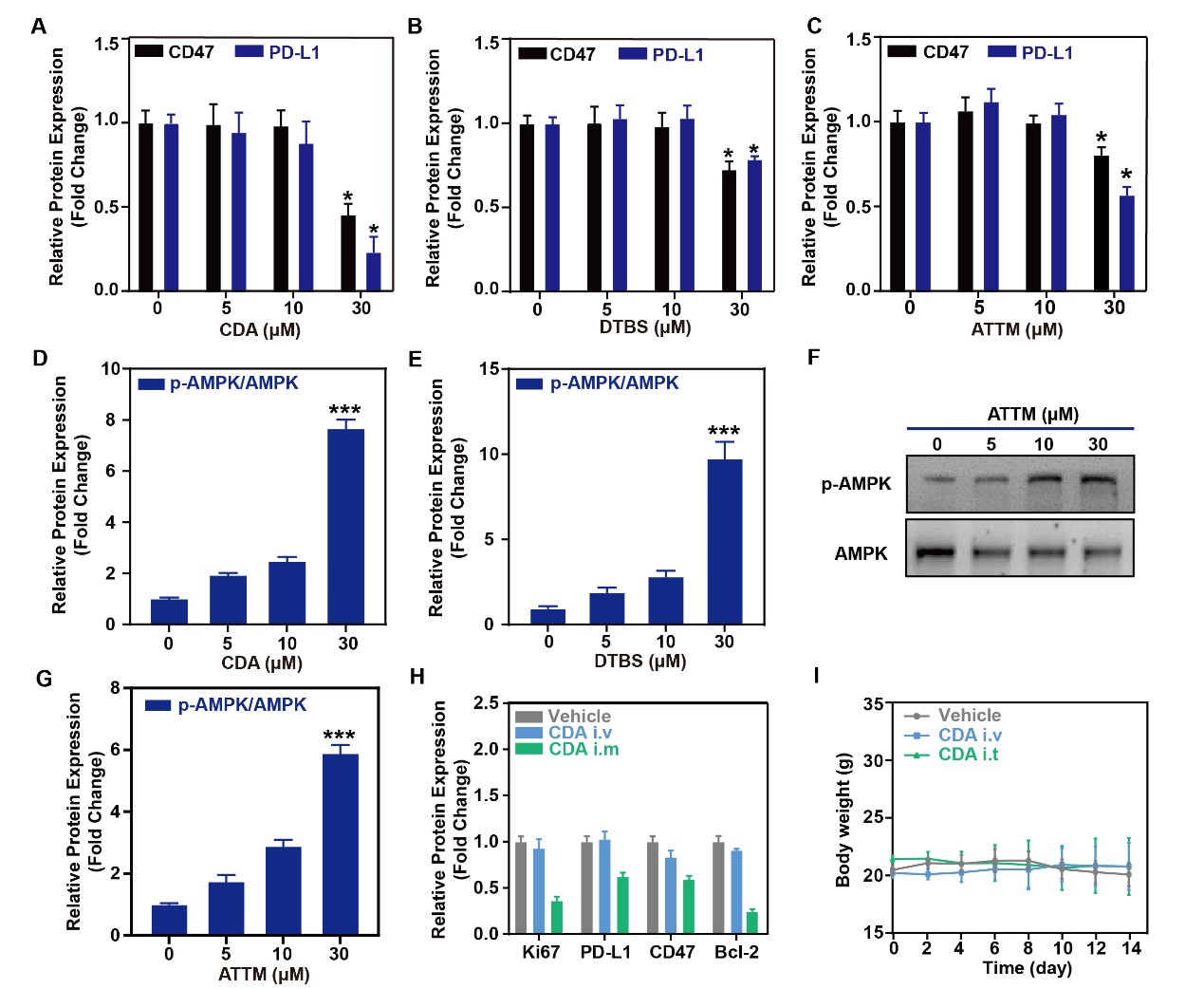


**Figure S6.** (A-C) Quantification of western blot grayscale intensity for Figure 1K (n = 3). (D-E) Quantification of western blot grayscale intensity for Figure 1L (n = 3). (F-G) Western blot analysis of AMPK and phosphorylated AMPK (p-AMPK) expression in MB49 cells cultured under copper-supplemented conditions and treated with different doses of ATTM (n = 3). (H) Quantification of western blot grayscale intensity for Figure 1M (n = 3). (I) Body weight monitoring of mice during CDA treatment in the *in vivo* tumor model (n = 5).


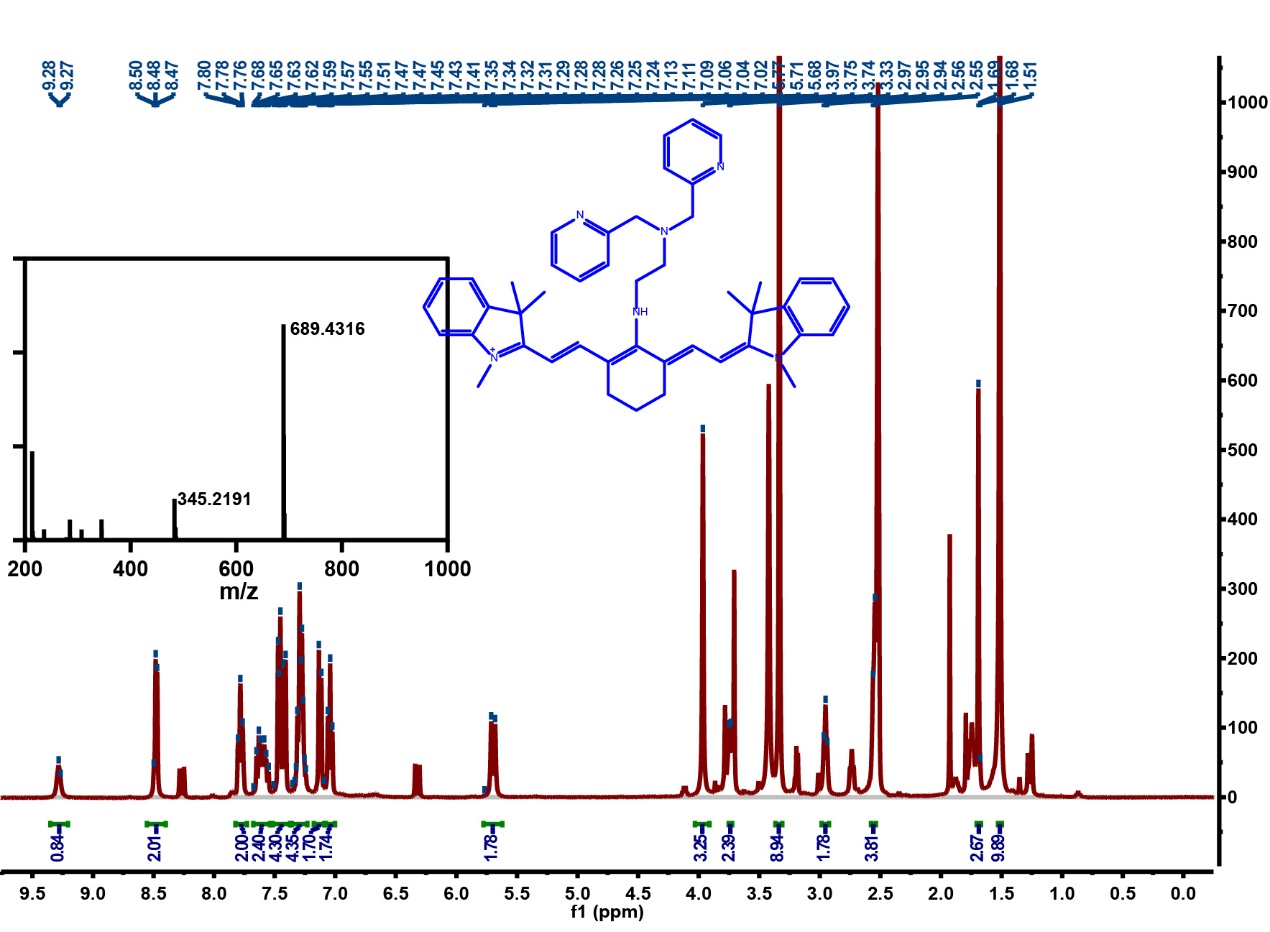


**Figure S7**. Verification of CYN-CDA by ^1^H-NMR and HR-MS.

**
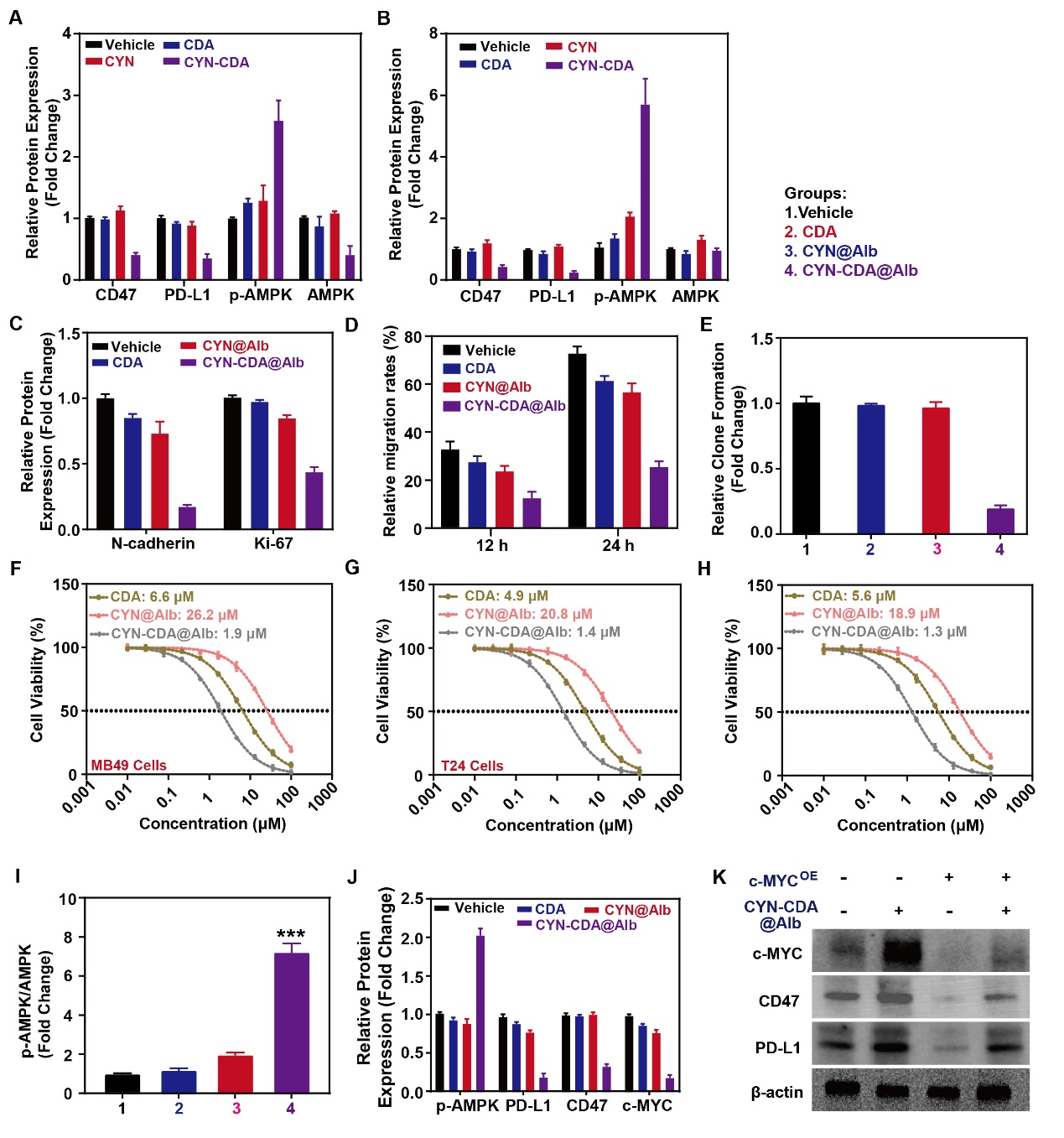
**

**Figure S8.** (A-B) Quantification of western blot grayscale intensity from Figure 2E-2F (n = 3). (C) Grayscale quantification of western blot bands shown in Figure 3F (n = 3). (D) Quantification of tumor cell migration capacity after different treatments, based on transwell or wound healing assay shown in Figure 3H. (E) Quantification of cell clone numbers in each treatment group from the colony formation assay shown in Figure 3G. (F-H) Determination of IC50 values of CDA, CYN@Alb, and CYN-CDA@Alb in MB49, T24, and 4T1 cells, respectively. (I) Grayscale quantification of western blot bands shown in Figure 3J (n = 3). (J) Western blot analysis of p-AMPK, PD-L1, CD47, and c-MYC expression levels in MB49 cells treated with different compounds, and corresponding grayscale quantification (n = 3). (K) Western blot analysis of CD47 and PD-L1 expression levels in MB49 cells or c-MYC over-expression MB49 cells treated with CYN-CDA@Alb.

**
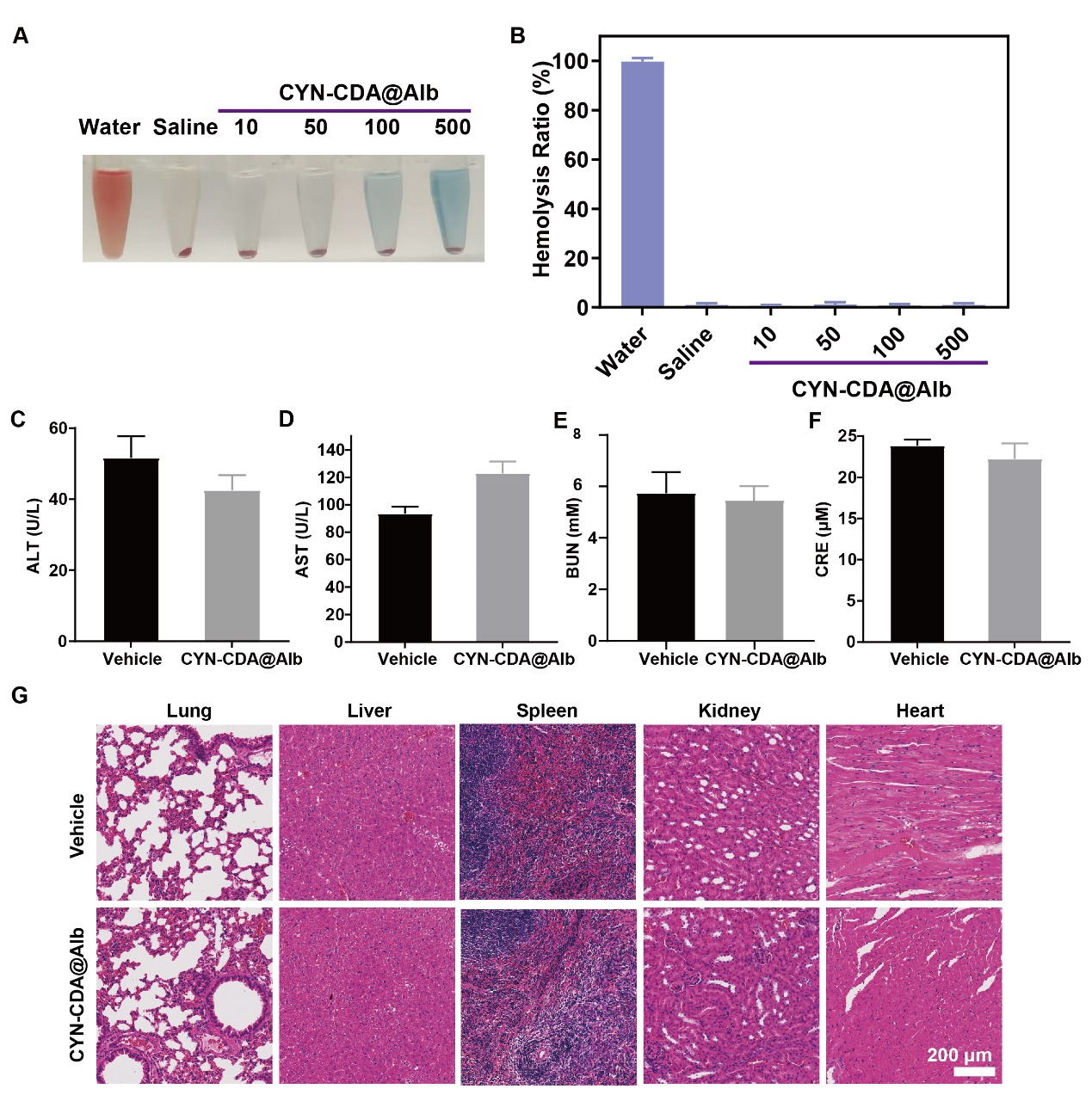
**

**Figure S9.** *In vivo* biosafety evaluation of CYN-CDA@Alb. (A-B) Hemolysis analysis of CYN-CDA@Alb. The percentage of hemolysis was quantified to evaluate blood compatibility. (C-F) Serum biochemical analysis of liver and kidney function (ALT, AST, BUN, and CRE) in mice treated with PBS or CYN-CDA@Alb (n = 5). (G) Representative H&E staining images of major organs (heart, liver, spleen, lung, and kidney) from mice treated with PBS or CYN-CDA@Alb.


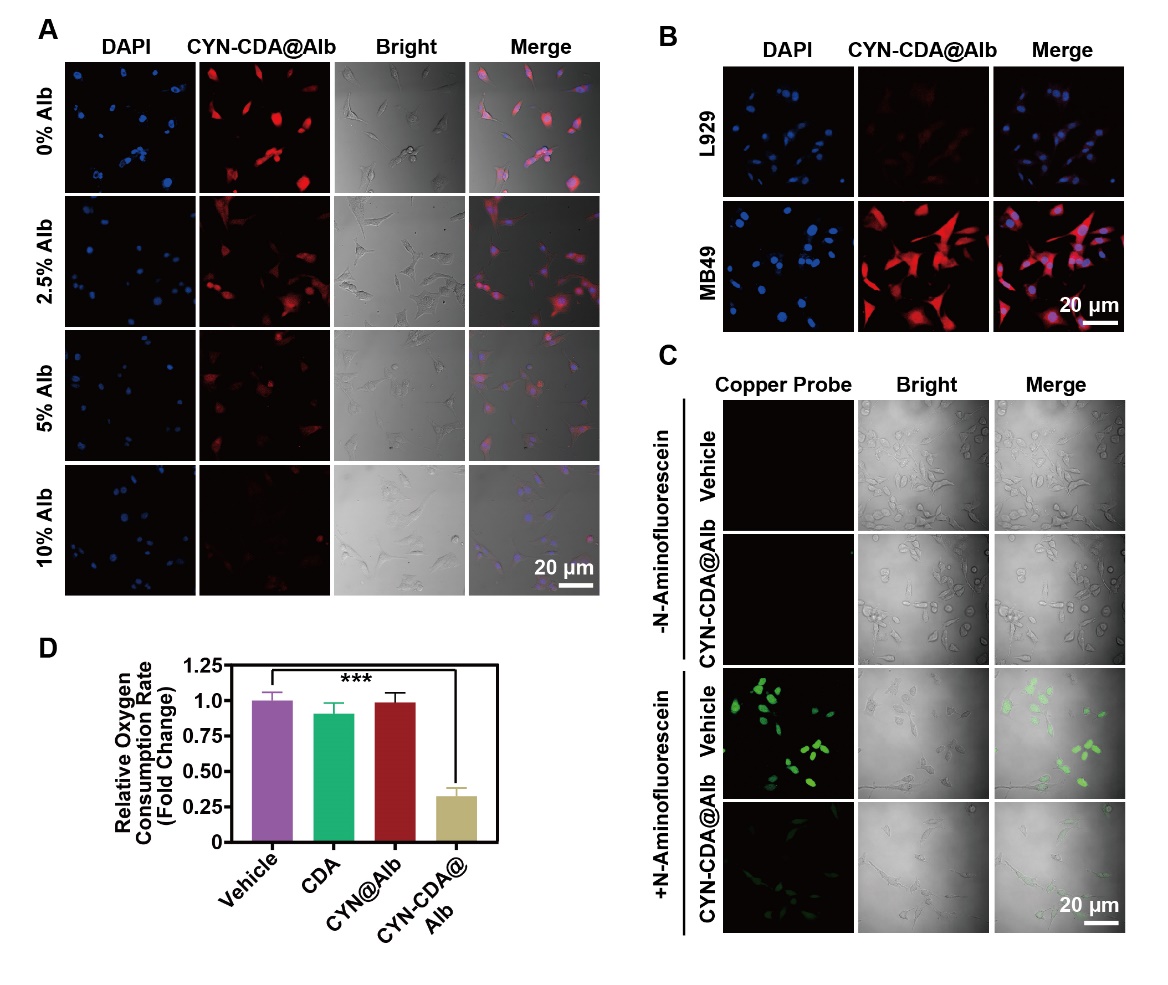


**Figure S10.** (A) Effects of Alb on the tumor cell accumulation behavior of CYN-CDA@Alb. (C) Efficacy of CYN-CDA@Alb-mediated copper disruption detected by copper probe N-Aminofluorescein. (D) Evaluation of the relative oxygen consumption rate (n = 3).

**
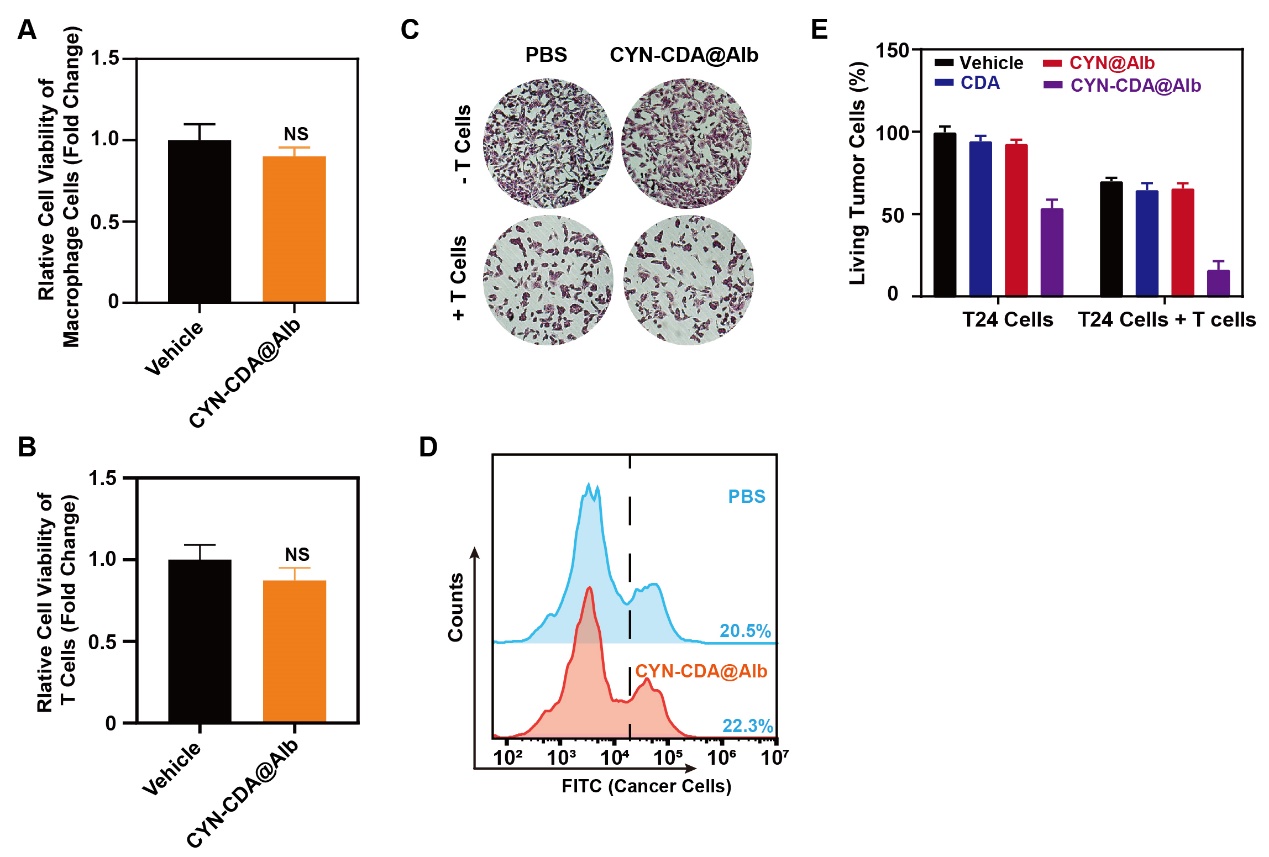
Figure S11.** (A-B) Effects of CYN-CDA@Alb on the cell viability of macrophage cells and T cells (n = 3). (C) Effects of the tumor cell killing viability of T cells. T cells were pre-treated with CYN-CDA@Alb (n = 3). (D) Effects of the tumor cell phagocytosis viability of macrophage cells. Macrophage cells were pre-treated with CYN-CDA@Alb (n = 3). (E) Quantification of residual tumor cell numbers after T cell-mediated killing assay as shown in Figure 3N (n = 3).

**
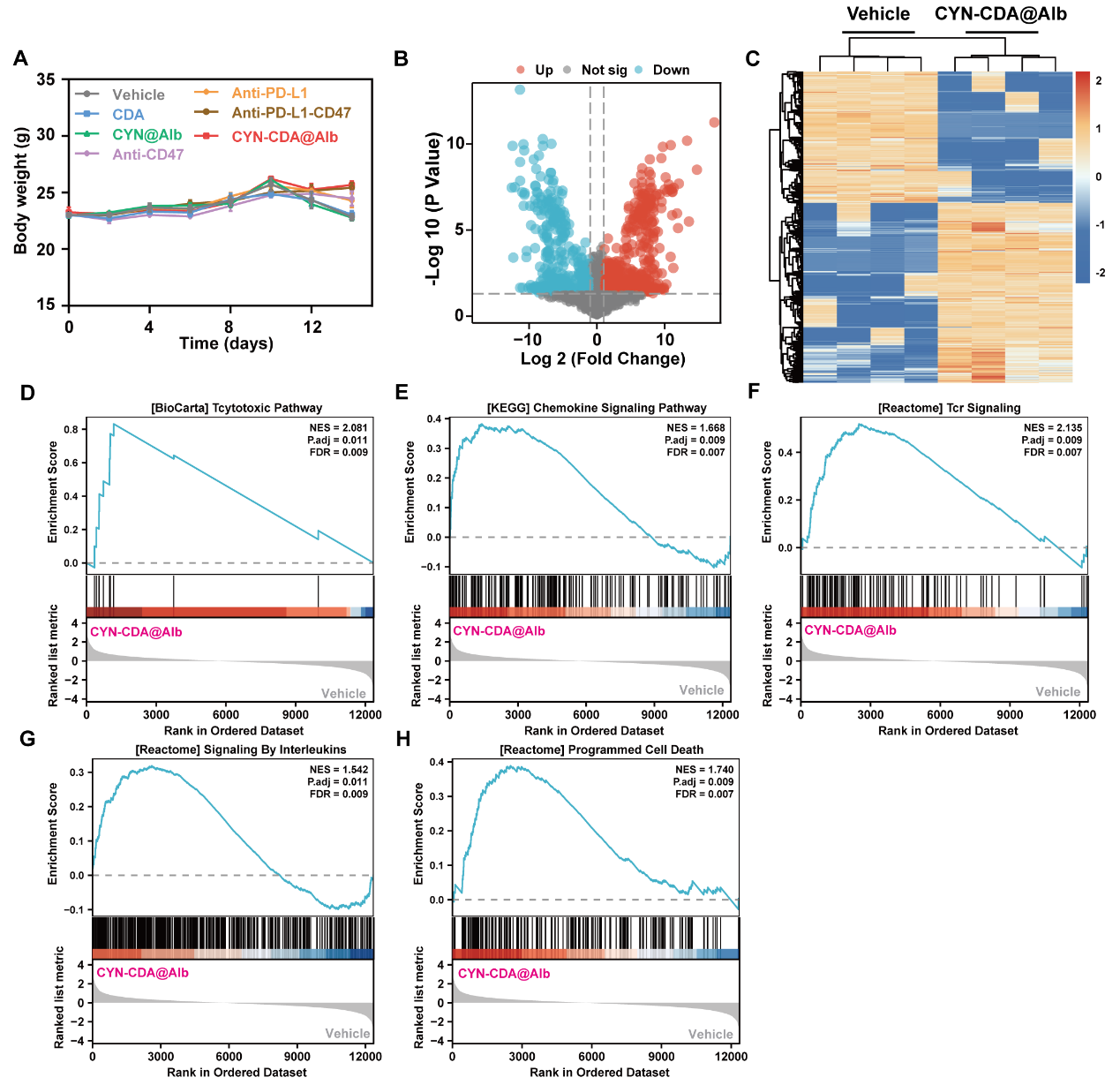
**

**Figure S12.** Antitumor immune response and proteomic profiling in CYN-CDA@Alb-treated mice. (A) Body weight curves of mice in the anti-tumor immunotherapy model (n = 5). (B) Volcano plot showing differentially expressed proteins in tumor tissues from PBS and CYN-CDA@Alb-treated mice based on proteomic analysis. (C) Heatmap illustrating hierarchical clustering of significantly altered proteins in tumor samples. (D-H) GSEA visualizations of proteomic data, highlighting key pathways modulated by CYN-CDA@Alb treatment.

**
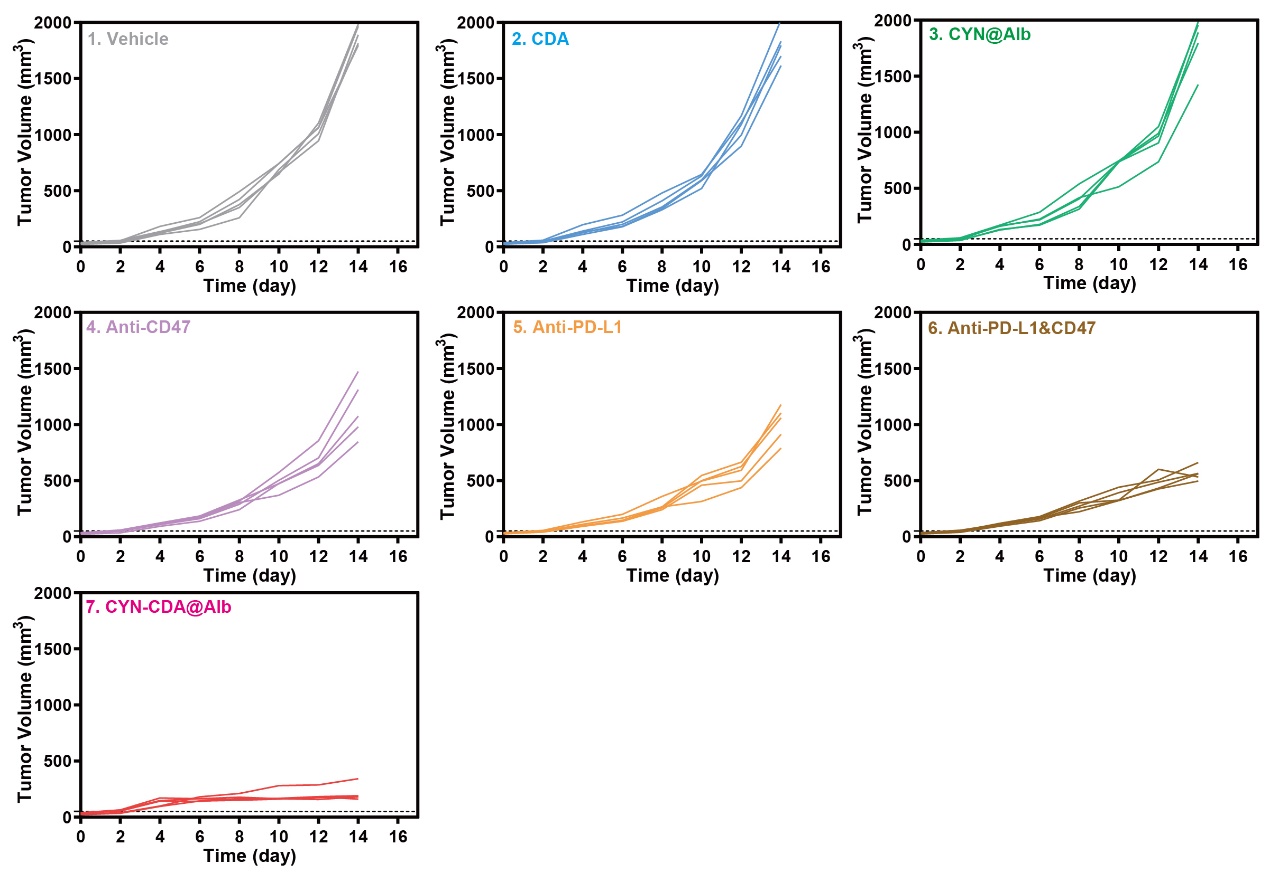
**

**Figure S13.** Curves of tumor volume changes in tumor-bearing mouse of each group in the mouse model

**
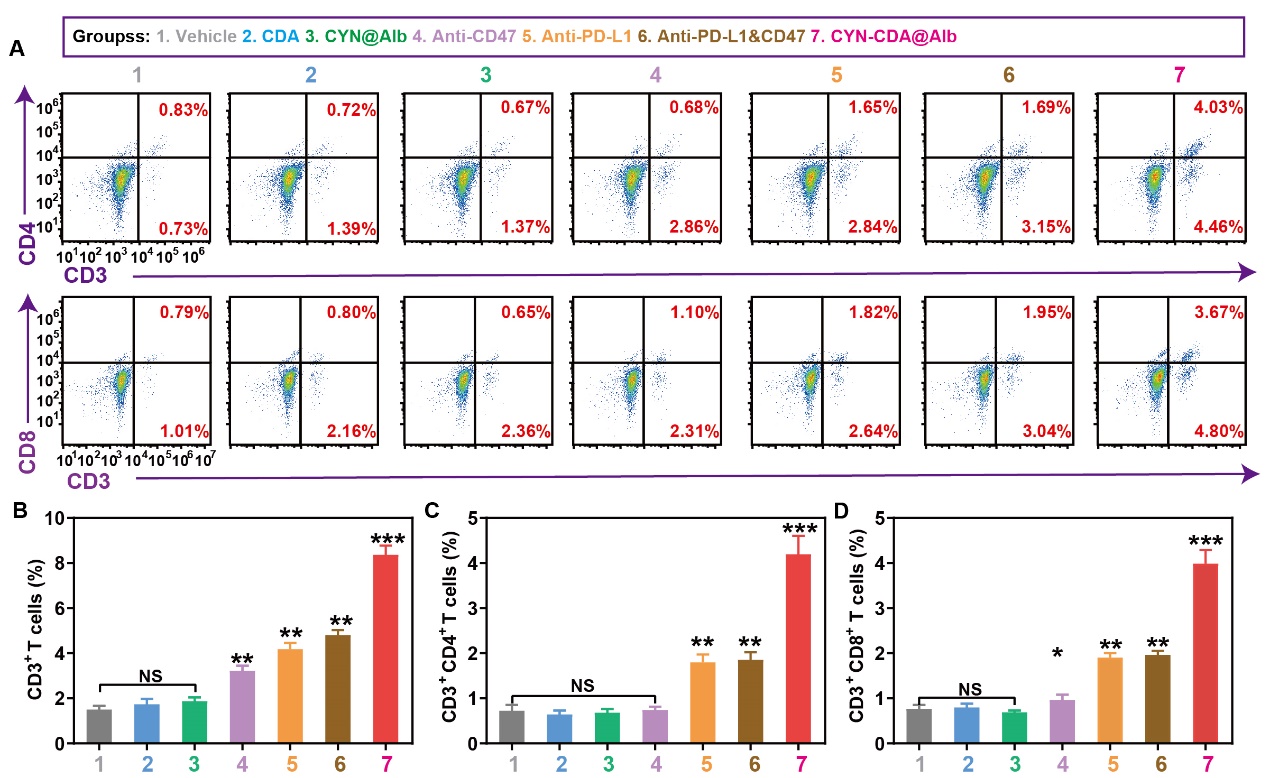
Figure S14.** (A-D) Flow cytometry analysis of tumor-infiltrating CD3⁺, CD4⁺, and CD8⁺ T cells in the anti-tumor immunotherapy model (n = 3), demonstrating enhanced T cell infiltration following CYN-CDA@Alb treatment.

**
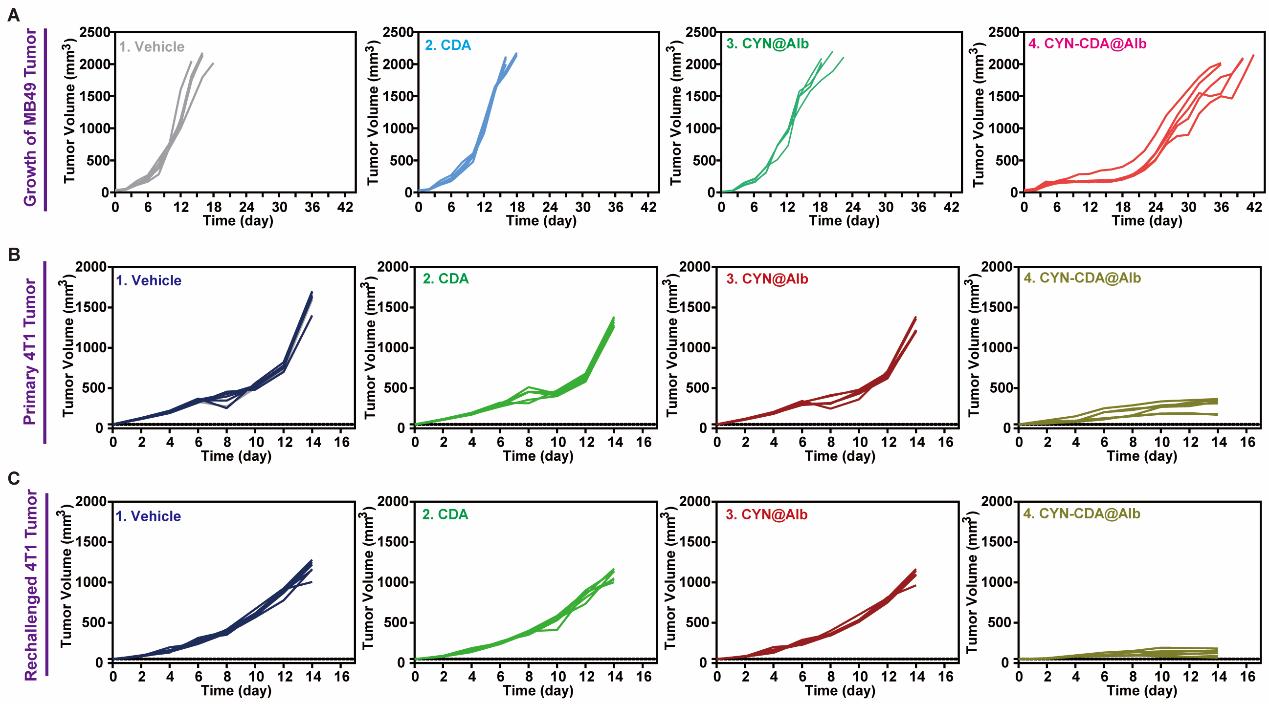
Figure S15.** (A) For the mouse model shown in Figure 4F, the tumor volume change curve of each tumor-bearing mouse in each group. (B) The mouse model shown in Figure 4I, the volume change curve of the subcutaneous tumor per tumor-bearing mouse of each group. (C) For the mouse model shown in Figure 4I, the distal tumor volume change curve of each tumor-bearing mouse in each group.

**
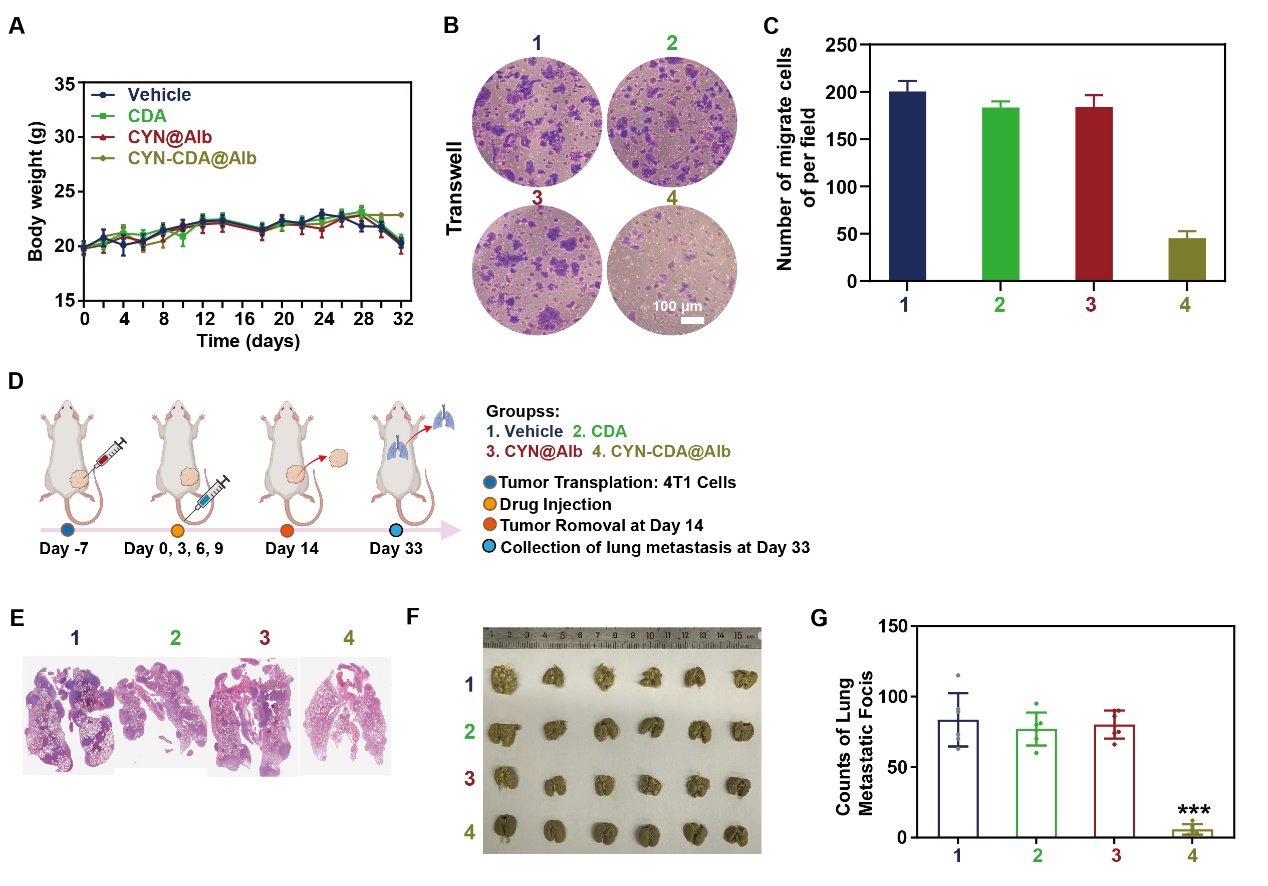
**

**Figure S16.** (A) The immune memory model in mice body weight change curve (n = 6). (B-C) Transwell assay quantification of invasive capacity of MB49 cells after different treatments (n = 3). (D) Schematic illustration of the lung metastasis model used to evaluate anti-metastatic efficacy *in vivo*. (E) Representative images of lung tissues and H&E staining from different treatment groups. (F-G) Lung tissue images and quantification of metastatic nodules in mice from each treatment group (n = 6).

**
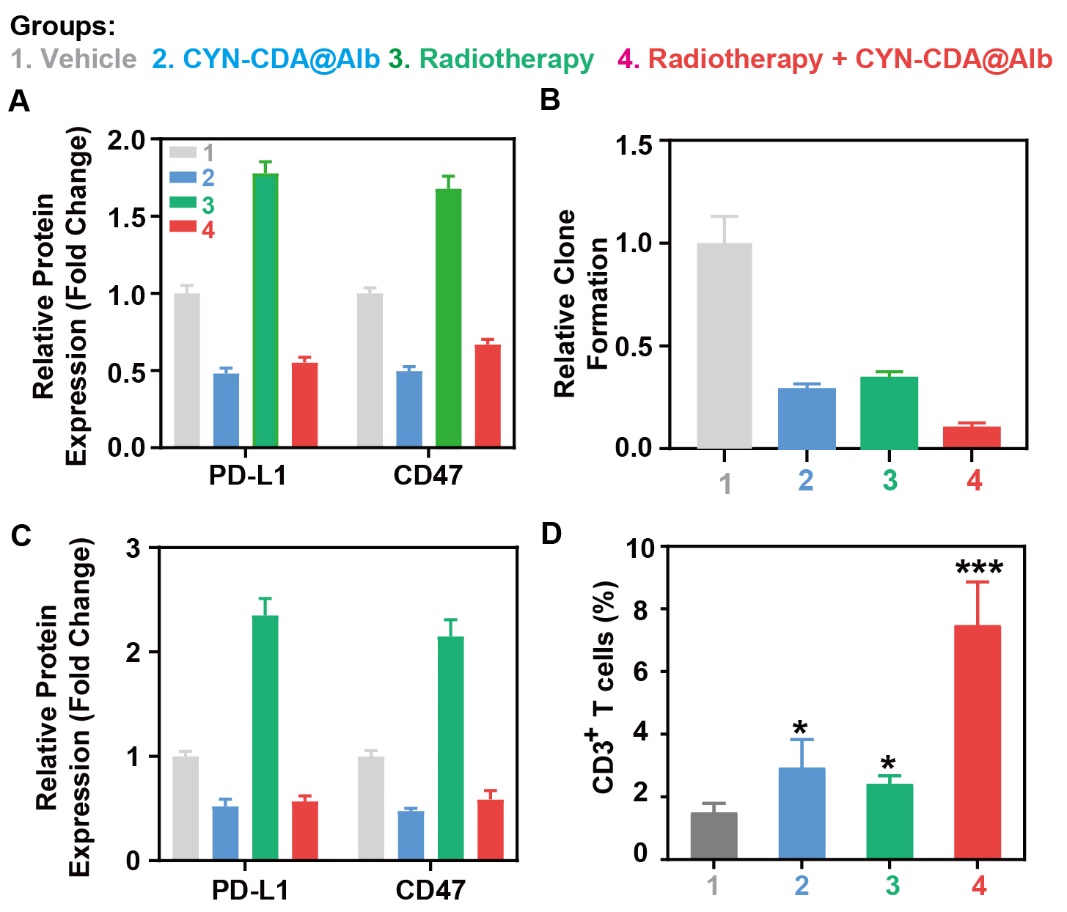
**

**Figure S17.** (A) Densitometric quantification of protein bands from Figure 5A (n = 3). (B) Quantification of colony numbers from the plate colony formation assay shown in Figure 5D under different treatment conditions (n = 3). (C) Densitometric analysis of western blot bands from Figure 5K (n = 3). (D) Quantification of CD3⁺ T cell infiltration based on flow cytometry results shown in Figure 5H.

**
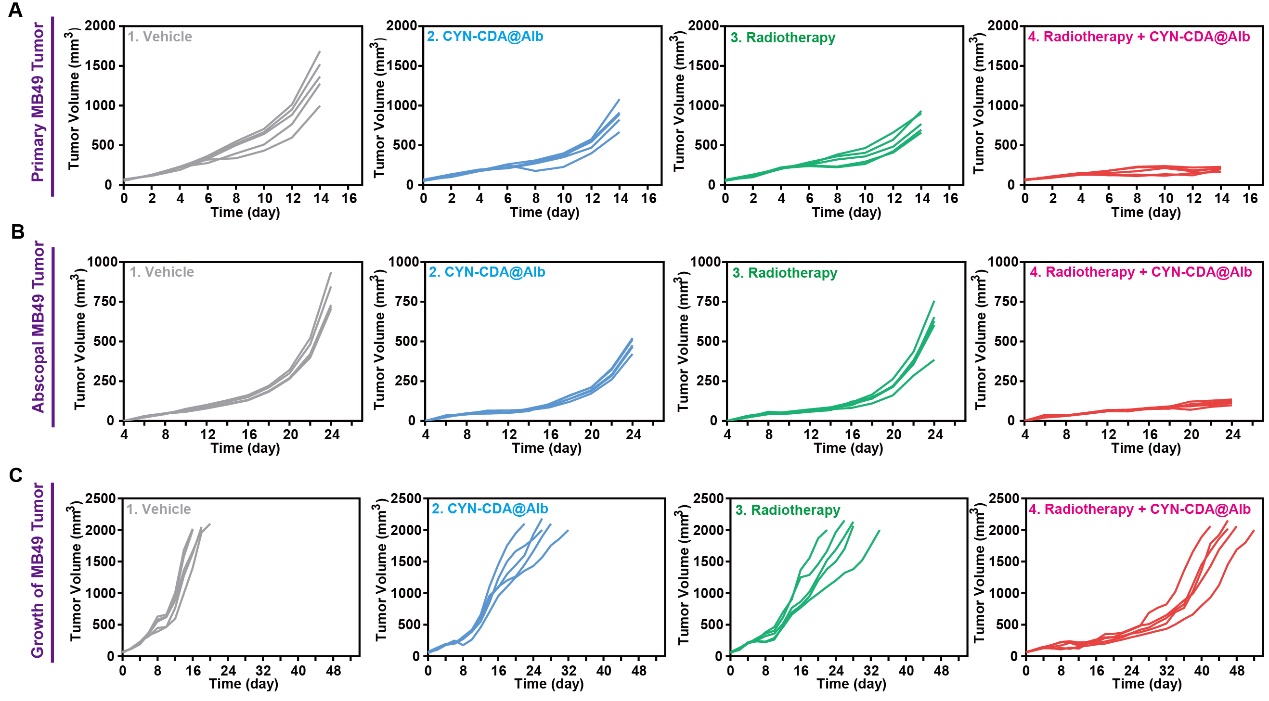
**

**Figure S18.** (A) The mouse model shown in Figure 5E, the primary tumor volume change curve of each tumor-bearing mouse in each group. (B) For the mouse model shown in Figure 5E, the volume change curve of the distal tumor of each tumor-bearing mouse in each group. (C) The mouse model shown in Figure 5O, the tumor volume change curve of each tumor-bearing mouse in each group.
